# Cotton Duplicated Genes Produced by Polyploidy Show Significantly Elevated and Unbalanced Evolutionary Rates, Overwhelmingly Perturbing Gene Tree Topology

**DOI:** 10.3389/fgene.2020.00239

**Published:** 2020-04-23

**Authors:** Fanbo Meng, Yuxin Pan, Jinpeng Wang, Jigao Yu, Chao Liu, Zhikang Zhang, Chendan Wei, He Guo, Xiyin Wang

**Affiliations:** ^1^School of Life Sciences, North China University of Science and Technology, Tangshan, China; ^2^Institute for Genomics and Bio-Big-Data, Chengdu University of Traditional Chinese Medicine, Chengdu, China

**Keywords:** cotton, gene collinearity, duplicated genes, orthologs, phylogeny, evolutionary rate, polyploidy

## Abstract

A phylogenetic tree can be used to illustrate the evolutionary relationship between a group of genes, especially duplicated genes, which are sources of genetic innovation and are often a hotspot of research. However, duplicated genes may have complex phylogenetic topologies due to changes in their evolutionary rates. Here, by constructing phylogenetic trees using different methods, we evaluated the phylogenetic relationships of duplicated genes produced by polyploidization in cotton. We found that at least 83.2% of phylogenetic trees did not conform the expected topology. Moreover, cotton homologous gene copy number has little effect on the topology of duplicated genes. Compared with their cacao orthologs, elevated evolutionary rates of cotton genes are responsible for distorted tree topology. Furthermore, as to both branch and site models, we inferred that positive natural selection during the divergence of fiber-development-related MYB genes was likely, and found that the reconstructed tree topology may often overestimate natural selection, as compared to the inference with the expected trees. Therefore, we emphasize the importance of borrowing precious information from gene collinearity in tree construction and evaluation, and have evidence to alert the citation of thousands of previous reports of adaptivity and functional innovation of duplicated genes.

## Introduction

Molecular phylogeny describes evolutionary relationships among organisms or genes that they harbor through molecular biology technology (Yang and Rannala, 2012). It is an area of molecular evolution and has attracted wide attention in recent years, mainly because it is difficult to evaluate phylogenetic relationship by any other method in many cases (Zhang et al., 2018). Molecular methods can provide much clearer answers to some long-standing phylogenetic problems than traditional methods. The accumulation of DNA sequence data has exerted a tremendous influence on the development of the phylogenetic system (Ma et al., 2017). The coming genome era provides even more opportunities to understand phylogenetic relationship among species and genes.

Phylogenetic trees are commonly used to illustrate the evolutionary relationship among a group of taxa. The order of species formation events leading to the formation of an extant taxonomic species is unique in history and is similar to the formation of an extant gene in a genome. Therefore, only one of all possible trees constructed with a given dataset can represent the true evolutionary history. The tree built from a specific set of data and a selected tree-building method may be the same as or different from the real tree. If a phylogenetic tree is made up of a gene from each species, the presumed tree is referred as a gene tree. It may differ from a species tree in topology or in branching pattern (Edwards, 2009; Jombart et al., 2017). The reasons may involve alternative loss of anciently duplicated homologs in each species or untoned evolutionary rates among homologs. If a phylogenetic tree is made up of multiple gene homologs from each and more species, often constructed to understand gene evolutionary trajectory or functional innovation, the tree could be much more complex and much diverted from the real tree, due to the above-mentioned reasons and more.

Commonly used tree-constructing methods can be divided into two types: distance-based method and character-based method. The former mainly includes unweighted pair group method with arithmetic means (UPGMA), weighted pair group method with arithmetic mean (WPGMA), neighbor-joining (NJ) (Saitou and Nei, 1987), least square (LS), and minimum evolution method (ME) (Edwards and CavalliS-forza, 1964). The latter includes maximum parsimony method (MP) (Burger, 1970; Fitch, 1971) and maximum likelihood method (ML) (Felsenstein, 1981; Yang, 1994).

Duplicated genes provide important opportunities for genetic novelty (van de Peer et al., 2009). It has been widely noted that multi-mer in a regulatory complex of proteins were likely produced by gene duplication. Multiple domains in a protein might also be made due to gene duplication. For example, disease resistance genes often have tens, or even hundreds, of copies in a genome, conferring resistance capability to fight fast evolving environmental pathogens. Therefore, the evolution and function of duplicated genes are a popular focus of interest in biology research. The existence of duplicated genes might provide more freedom to them, resulting in elevated or unbalanced evolutionary rates and functional innovation (Lynch and Conery, 2000; Salman-Minkov et al., 2016). During evolution, two homologs derived from a common ancestral gene may each partially take the ancestor’s functions, often with multiple functions conflicting in time and space in cells, resulting in sub-functionalization (Lynch and Force, 2000); one homologous copy may evolve new function(s), referred as neo-functionalization. A combination of the above functional innovations could also occur, or one copy can be pseudogenized or lost to lower functional redundancy or to eliminate dosage changes, resulting in non-functionalization.

In a plant genome, there are often thousands of duplicated genes produced by various means of genetic duplication, such as polyploidization, tandem gene duplication, or transposon-related duplication, etc. Recursive polyploidization specifically has made plant genomes very complex and provides a great impact on evolutionary and functional innovation (Charon et al., 2012; Kim et al., 2014; Liu et al., 2014). Though wide-spread gene losses often occurred, a polyploidization event often produced thousands of duplicated genes in an extant plant genome. Major eudicot plants share a hexaploid ancestor, and the corresponding event has around 1600 duplicated copies in an eudicot plant, such as grape, cotton, soybean, etc. (Jaillon et al., 2007; Jiao et al., 2011; Wang et al., 2017). Cotton was affected by a decaploidization event, likely shared with the other Gossypium relatives, but not the non-Gossypium Malvaceae plants, such as durian and cacao (Wang et al., 2016, 2019). This means that, if no gene loss had occurred, a grape gene would have one cacao ortholog and five orthologs in collinear positions in *Gossypium raimondii* (DD). However, owing to wide-spread gene losses after the decaploidization, only 39.1% of cacao genes have two or more duplicated copies at colinear positions in extant cotton DD genome (Wang et al., 2016). The inference of these thousands of years of polyploidization events and their produced duplicated genes were based on the detection of gene collinearity between chromosomes, and a set of collinear homologs were supposed to be derived from a common ancestral gene in the genome before the decaploidization.

Phylogenetic and evolutionary analysis of plant genomes may be problematic due to the changes in their evolutionary rates after polyploidiztion. In Gramineae, it has been suggested that barley (*Hordeum vulgare*), sorghum (*Sorghum bicolor*), and maize (*Zea mays*) evolve 12–33% faster than rice (*Oryza sativa*), which retains the most conserved genome (Wang et al., 2015). Assuming that genes evolve at the same rate will lead to weird inferences when duplicate genes in different species are used to determine the age of the same polyploidization. Using grape orthologs as a reference, the comparison of cacao and cotton genes showed that cotton genes evolved 19 and 15% faster at synonymous and non-synonymous substitution sites than their cacao homologs, respectively. Cacao was used to assess the difference in evolutionary rates between durian and cotton, indicating that after division from cacao, cotton evolves about 64% faster than durian (Wang et al., 2019). The difference between cotton paralogs is greater than the difference between them and cacao orthologs due to the increased rate of cotton evolution. The higher evolutionary rate in cotton than its related species was at least partly attributed to the occurrence of the polyploidization in cotton, as an elevated evolutionary rate of genes was also observed in other paleopolyploidies (Wang et al., 2011; The Tomato Genome Consortium., 2012; Chalhoub et al., 2014).

Elevated evolutionary rates of cotton genes raised the following interesting questions: how have the phylogeny of duplicated homolog genes that produced simultaneously been affected? Do the trees conform to the real or expected gene trees? What can be understood from the unbalanced nature of the evolution of the duplicated genes? Is there any difference from the large gene families? Is there evidence of adaptive selection and how does diverted phylogeny affect the inference of adaptive evolution? To answer the above questions, here, we used different tree-constructing methods to build phylogenetic trees of colinear homologous genes in collinearity between genomes of cotton, grape, and cacao, analyzed the topological structure of homologous gene trees, compared them with the expected trees inferred based on gene collinearity, and assessed the difference in inferring selective pressure based on the constructed and the expected trees.

## Materials and Methods

### Materials

Genome data were retrieved from public databases: grape (*Vitis vinifera*; v12X) and cotton DD (*Gossypium raimondii*) genomes from phytozome (v2.1)^1^, allotetraploid cotton AADD (*Gossypium hirsutum* and *Gossypium barbadense*) genomes and cotton AA (*Gossypium arboreum*) genome from Cottongen^2^ (Yu et al., 2014), and cacao (*Theobroma cacao*) genome (v2) from CocogenDB^3^. Alignment of collinear genes including grape, cacao, and cotton were downloaded from previous reports^4^; an updated version of grape genes was related to the information of gene collinearity.

### Gene Phylogeny Construction

The coding sequences (CDS) of each group of genes were aligned using ClustalW (Thompson et al., 1994; Larkin et al., 2007) and MUSCLE (Edgar, 2004), and tree construction was performed using five methods in MEGA X (Kumar et al., 2018) (Maximum Likelihood, Neighbor-Joining, Minimum-Evolution, UPGMA, and Maximum Parsimony) with default parameters. Grape was set as the outgroup for each phylogenetic tree. The reliability of an inferred tree was characterized with bootstrap analysis with 1000 replications.

### Gene Copy Number Analysis

BLASTP (Camacho et al., 2009) was used to align protein sequences of grape with cotton to detect the copy number of each grape gene in the cotton. We characterized gene copy variation in cotton by searching each grape gene at two *E*-value cutoffs of 1e−5 and 1e−20, respectively.

### Calculation of Ks

Synonymous nucleotide substitutions on synonymous sites (Ks) were estimated by using the Nei-Gojobori approach (Nei and Gojobori, 1986) by implementing the Bioperl Statistical module.

### Selective Pressure Detection

MYB family transcription factor genes were identified in diploid and tetraploid cotton genomes using a list of seed genes retrieved from a previous report (Paterson et al., 2012). With MYB genes, PhyML 3.0 was used to build ML trees, with the Jones, Taylor, and Thorton (JTT) model and 100 non-parametric bootstrap replicates (Guindon et al., 2010). We applied likelihood ratio (LR) tests to detect likely positive selection based on the ML methods and codon substitution models. Based on previously reported methods (Mondragon-Palomino et al., 2002; Mondragon-Palomino and Gaut, 2005), we implemented Codeml from the PAML package and analyzed the ML and expected trees to infer ω, the ratio of the non-synonymous to synonymous distances (Yang, 1997; Yang et al., 2000). We detected variation in ω among sites by employing a likelihood ratio test between M0 and M1, and M7 and M8 models.

## Results

### Homologous Gene Phylogenetic Tree

**FIGURE 1:**
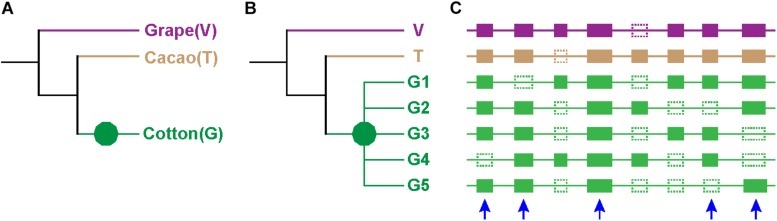
Expected tree topology. **(A)** Phylogeny of cotton (G), cacao (T), and grape (V). Different branches are in different colors. **(B)** Phylogenetic trees of genes: green decagon denotes decaploidization of cotton. The group of cotton genes G1, G2, G3, G4, and G5 are paralogous to one another, and they have one grape and one cacao ortholog (V and T). **(C)** Homologs at the collinear positions of the three genomes: solid rectangle denotes genes, and dotted ones denotes lost genes. Genes from different species are shown in different colors, and the color scheme is consistent to those in **(A,B)**. The blue arrow denotes the selected homolog.

To understand the evolution of cotton’s duplicated genes, we involved cacao, the close relative of cotton, and grape, with a genome closely resembling that of the ancestral eudicot, to our experience in plant genomics analysis, in the present analysis. We selected 662 groups of homologs at the collinear positions of the three genomes involved, and each group had one grape gene as the outgroup of other homologs, one cacao gene, and at least three colinear cotton paralogs, being orthologous to the grape and the cacao gene (Figure 1 and Supplementary Table S1). The cotton paralogs were likely produced by the decaploidization. 2123 cotton genes were involved in the above homologous groups, i.e., each group has an average 3.21 cotton paralogs. Fractions of 81.4, 16.5, and 2.1% of all the homologous groups have 3, 4, or 5 cotton paralogs, respectively.

**FIGURE 2:**
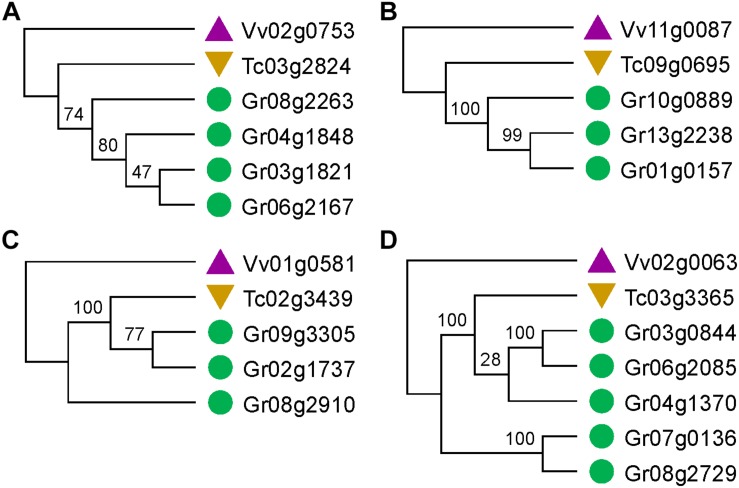
Gene trees that match or not match the expected topology. **(A,B)** Gene trees that match the expected topology. **(C,D)** Gene trees that do not match the expected topology.

Notably, only a quite small fraction of trees conformed to the expected tree topology (Figure 1, Table 1, and Supplementary Table S2). The expected trees reflect the relationship of colinear paralogs produced by the decaploidy, and colinear orthologs originated due to speciation (Figure 1). That is, the decaploidy-produced cotton paralogs were expected to group together, with the cacao and the grape orthologs being their outgroup. Coding sequences of genes were translated into amino acid sequences to produce the alignment. ClustalW was used to make the alignment. With four tree-constructing approaches, ML, NJ, ME, and MP, an average 15.2% of constructed trees agree with the expected tree topology (Table 1). With the ML approach, a maximum of 16.8% of constructed trees agree with the expected tree. In contrast, with a UPGMA approach, a minimum of 0.9% of trees matched the expected topology, showing that it is the weakest choice to reconstruct the right phylogeny. With all approaches, only five (0.76%) groups of homologs have all constructed trees conforming to the expected topology, while 503 (76%) groups have all trees not conforming to the expected topology (Figure 2). With the non-UPGMA approaches, only 45 (6.8%) groups of homologs all have constructed gene trees conforming to the expected topology, while 503 (76%) groups all have trees not conforming to the expected topology.

**TABLE 1:** Number of gene trees conforming to the expected topology using different methods after aligned by ClustalW.

**Method**	**Number**	**Percentage**
Maximum likelihood	111	16.8%
Neighbor-joining	97	14.7%
Minimum-evolution	106	16.0%
UPGMA	6	0.9%
Maximum parsimony	87	13.1%

Different alignment approaches did not affect the tree topology much. Sequences were also aligned using MUSCLE and then trees were constructed with the above five approaches (Supplementary Tables S2, S3). Results were very similar compared to the trees constructed using ClustalW. One thing to note is that with the MP approach, the trees constructed with MUSCLE had ∼2% increase agreeing with the expected topology as compared to those constructed with ClustalW.

### Gene Homologous Copy Number and Tree Topology

Besides collinear homologs, there are other homologs in the cotton genome which increase redundancy. And these homologs, which are theoretically likely to elevate evolutionary freedom, possibly affect the topology of the trees constructed above. Here, for each contrasted tree, we checked whether the cotton collinear homologs have other homologous copies in the whole genome. Initially, at the BLASTP *e*-value < 1e−20, we searched homologous copies, and grossly did not find much relationship between cotton homologous copy numbers and the percentages of trees agreeing with the expected topology. However, when homologous copy number < 5, the agreeing percentage reached its maximum (21.3%) with the ML approach (Table 2). In consideration of a much divergent and fast evolutionary rates of genes, especially when they are tens of millions of years old as is the occurrence of the decaploidy, we lowered the criteria to find homologs to BLASTP *e*-value < 1e−5, for three tree-constructing approaches (ML, NJ, and ME), when homologous copy number < 5, the agreeing percentages reaches the highest values (22.2%) (Supplementary Table S4); and in contrast, when copy number ≥ 50, the agreeing percentages reaches the lowest values (10.1%) (Supplementary Table S4).

**TABLE 2:** Proportion of gene trees with expected topologies over different copy number ranges (*E-*value cutoff of 1e−20).

**Method**	**Range of copy numbers**				
	**[0, 5)**	**[5, 10)**	**[10, 20)**	**[20, 50)**	**[50, 3113)**
Maximum likelihood	21.3%	14.0%	19.9%	12.1%	18.5%
Neighbor-joining	18.1%	10.5%	20.5%	11.4%	12.3%
Minimum-evolution	18.1%	14.0%	21.0%	12.9%	12.3%
UPGMA	1.1%	0.6%	1.1%	1.4%	0.0%
Maximum Parsimony	14.9%	9.9%	17.0%	11.4%	12.3%

### Elevated Evolutionary Rates of Cotton Homologs

Elevated evolutionary rates of cotton genes are responsible for distorted tree topology. In all the distorted trees, the cacao gene is clustered with one or more cotton genes with the other cotton homolog(s) to form their outgroup branch(es) (Figure 2). This finding could be explained by elevated evolutionary rates of those cotton genes forming outgroup branches. Here, by estimating synonymous nucleotide substitutions at the synonymous site (Ks), we characterized the level of difference between cotton-grape and cotton-cacao homologous genes (Table 3). To find whether numbers of cotton paralogs affect their evolutionary rates, we checked 3803 homologous gene groups, including those with one and two cotton colinear paralogs, which were previously excluded in tree construction analysis. We found that, with groups with 1–4 cotton homologs, the average of Ks between cotton and grape orthologs is significantly larger than the Ks between cacao and grape orthologs (*T*-test *P*-value ≤ 0.008).

**TABLE 3:** Differences in Ks between homologous pairs of different species.

	**1Gr**	**2Gr**	**3Gr**	**4Gr**	**5Gr**
Numbers	1505	1649	527	109	13
Average	17.1%	17.4%	18.4%	14.1%	9.1%
Max	17.1%	38.6%	53.3%	59.5%	53.3%
Min	17.1%	−3.8%	−16.0%	−31.6%	−33.5%

The copy number of collinear cotton paralogs may affect their evolutionary rates. To characterize how elevated cotton genes were in evolutionary rates as to their respective cacao orthologs, we defined the average relative rate difference ratio, *R_A_* = [Average(Gr_i_Vv)−TcVv]/TcVv, where GrVv is the Ks between a cotton gene and its grape ortholog, and TcVv is the Ks between the cacao gene and its grape ortholog, and average Ks were found when multiple cotton colinear genes were involved. As to the *R*_A_, we found that cotton genes evolved 9.1–18.4% (significantly) faster than their cacao orthologs with different groups involving 2–5 cotton colinear paralogs (Table 3). Actually, even when there is only one cotton colinear gene preserved, the cotton-grape orthologs’ Ks is significantly larger than the cacao-grape orthologs’ Ks values (*T*-test *P*-value = 2.32e−11).

Moreover, cotton homologs showed significantly diverged evolutionary rates. While there are cotton homologs that have much higher evolutionary rates, in the meantime, there are ones that have evolved even slower than their cacao orthologs. We defined the maximal and minimal relative rate difference ratios,

R=Max[Max(GrVvi)-TcVv]/TcVv

and

R=Min[Min(GriVv)-TcVv]/TcVv

where the maximal and minimal Ks were found when multiple cotton colinear paralogs were involved. When there are two cotton colinear paralogs, as to *R*_Max_ and *R*_Min_, one cotton gene evolved 38.6% faster than its cacao ortholog (Table 3), while the other cotton gene evolved 3.8% slower than the cacao ortholog. More divergent evolutionary rates were found with the groups with more cotton paralogs. The cotton genes having evolved much faster are more likely to develop new functions, i.e., neofunctionalization, while the ones much slower are more likely to preserve the ancestral function(s).

### Problematic Tree Topology Affects Inference of Natural Selection

Of the 662 groups of homologous genes selected, 13 groups of them contain MYB family transcription factor genes, which may help differentiate epidermal cells into fibers. To understand whether problematic tree topology affected the assessment of functional innovation, we detected the orthologous genes from the cultivated cottons, *G. hirsutum*, *G. barbadense*, and *G. arboretum*. A total of 199 MYB family transcription factor gene homologs were included in the following analysis, with an average of 15.3 per group.

We performed natural selection pressure analyses on the constructed ML trees and the expected trees, respectively. The expected trees were made as to the relationship of colinear homologs, as described above (Figure 1). Notably, we found that none of the trees constructed by the ML method conformed to the expected topology. As to the branch model, only one group inferred the same gene homologs to be positively selected, ignoring the difference in ω values among branches leading to the gene homologs on the reconstructed and the expected trees.

**FIGURE 3:**
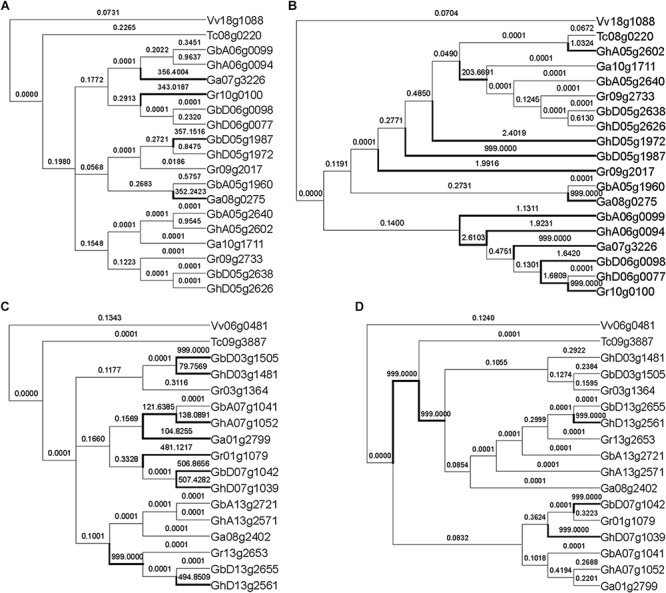
Natural selection inference of MYB genes with expected tree and reconstructed tree. **(A)** Inference of natural selection of RAD-like genes with the expected topology. **(B)** Inference of natural selection of RAD-like genes with the reconstructed topology. **(C)** Inference of natural selection of MYB59 genes with the expected topology. **(D)** Inference of natural selection of MYB59 genes with the reconstructed topology. The number on the branch is the ω value, and the branch with ω > 1 is marked with a thick line.

With the other 12 groups of MYB genes, the reconstructed and the expected trees came to very different inferences of positively selected genes (Figure 3 and Supplementary Figure S1). Often (8/12) the expected trees inferred fewer positively selected genes or branches. As to the reconstructed tree, a RAD-like gene inferred 12 positively selected branches or genes, as compared to only 4 in the expected tree, showing much more over-assessment of natural selection (Figures 3A,B). Surely, the tree topology difference should be responsible of this over-assessment. Checking the tree topology, we found that one cotton gene GhA05g2602 was clustered together with the cacao ortholog, Tc08g0220, while the other cotton genes were their outgroup. This shows that GhA05g2602 may have preserved ancestral gene function, while the other cotton gene had elevated evolutionary rates. In contrast, a MYB59 gene inferred ten positively selected branches and genes with the expected tree and more than five with the reconstructed tree (Figures 3C,D). It seems that one of three sets of decaploidy-derived colinear cotton paralogs elevated the evolutionary rates and moved outsides of the branch of the cacao ortholog, Tc09g3887.

The reconstructed and the expected trees often inferred different positively selected sites. As to the site model using both NEB (naïve empirical Bayes) and BEB (Bayes empirical Bayes) posteria tests, there were only 4 out of 13 groups inferring all the same positive selection sites with both the expected and the reconstructed trees (Supplementary Table S5). However, only two sites had a posterior probability greater than 0.95, verified by NEB and BEB.

## Discussion

The selected 662 colinear homologous groups described above provided a valuable evaluation as to whether correct trees could be produced based on sequence alignment. As we have shown, the majority (80%) of reconstructed trees could not reflect the actual relationship between these homologs. Without a reliable tree reflecting the actual relationship of their evolutionary history, one cannot rightly infer evolutionary innovation of genes. Here, we showed that nearly all cases that infer positive selection are not right.

Fortunately, gene collinearity within a genome and between genomes could, to some extent, make up the flaws in tree topology reconstruction. Colinear genes often have a deterministic origination. Within a genome, they may be produced by rounds of polyploidization, and each of the polyploidization events are often well inferred about their occurrence time and related ploidy number. Between two genomes, colinear genes are often credible orthologs due to speciation, or outparalogs produced by polyploidization affected their common ancestor. Here, the above analysis involved a major-eudicot-common hexaploidy, and a decaploidy specific to the cotton lineage. The homologs produced by these two events were well grouped into orthologs and (out)paralogs. Therefore, without sequence alignment and tree reconstruction, we know their actual relationship used to construct the expected trees. With these expected trees, we could perform correct evolutionary analysis. To our knowledge, this is the first time gene collinearity is emphasized to be introduced to gene tree construction and evolutionary analysis of duplicated genes.

Some may doubt that alternative loss of anciently duplicated genes may result in problematic evaluation. This likelihood was lowered to its utmost with the cross-multigenomic collinearity restriction. Ancestral duplication at chromosomal location might result in tandem genes. In fact, only a small fraction (8.7%) of these genes have a tandem homology in at least a considered genome. Therefore, the likelihood of a tree topology resulted from the alternative loss of ancestral genes is very slim.

Many and many trees have been constructed to infer and describe their evolutionary history and functional innovation. Duplicated genes are the key to understanding gene functional innovation. However, without gene collinearity support, we have reasons to worry whether this research could have come to the right conclusion, and would advise double checking their reports of adaptivity and functional innovations of genes or encoded proteins.

## Data Availability Statement

The raw data supporting the conclusions of this article will be made available by the authors, without undue reservation, to any qualified researcher.

## Author Contributions

XW conceived and led the research. FM, YP, and JW performed the analysis. JY, CL, ZZ, CW, and HG contributed to data collection and joined constructive discussions. XW, YP, and JW wrote the manuscript.

## Conflict of Interest

The authors declare that the research was conducted in the absence of any commercial or financial relationships that could be construed as a potential conflict of interest.

## Footnotes

1 https://phytozome.jgi.doe.gov/

2 https://www.cottongen.org/

3 http://cacaogendb.cirad.fr/

4 https://doi.org/10.1111/nph.13689

## References

[B1] Burger A. (1970). Atlas of protein sequence and structure 1969. *J. Med. Chem.* 13:337 10.1021/jm00296a903

[B2] Camacho C. Coulouris G. Avagyan V. Ma N. Papadopoulos J. Bealer K. (2009). BLAST+: architecture and applications. *BMC Bioinformatics* 10:421. 10.1186/1471-2105-10-421 20003500 PMC2803857

[B3] Chalhoub B. Denoeud F. Liu S. Parkin I. A. P. Tang H. Wang X. (2014). Plant genetics. Early allopolyploid evolution in the post-Neolithic *Brassica napus* oilseed genome. *Science* 345 950–953. 10.1126/science.1253435 25146293

[B4] Charon C. Bruggeman Q. Thareau V. Henry Y. (2012). Gene duplication within the Green Lineage: the case of *TEL* genes. *J. Exp. Bot.* 63 5061–5077. 10.1093/jxb/ers181 22865910

[B5] Edgar R. C. (2004). MUSCLE: a multiple sequence alignment method with reduced time and space complexity. *BMC Bioinformatics* 5:113. 10.1186/1471-2105-5-113 15318951 PMC517706

[B6] Edwards A. W. F. CavalliSforza L. L. (1964). “Reconstruction of evolutionary trees,” in *Phenetic and Phylogenetic Classification*, eds Heywood V. H. McNeill J. (London: The Systematics Association) 67–76.

[B7] Edwards S. V. (2009). Is a new and general theory of molecular systematics emerging? *Evolution* 63 1–19. 10.1111/j.1558-5646.2008.00549.x 19146594

[B8] Felsenstein J. (1981). Evolutionary trees from DNA sequences: a maximum likelihood approach. *J. Mol. Evol.* 17 368–376. 10.1007/bf01734359 7288891

[B9] Fitch W. M. (1971). Toward defining the course of evolution: minimum change for a specific tree topology. *Syst. Biol.* 20 406–416. 10.1093/sysbio/20.4.406

[B10] Guindon S. Dufayard J.-F. Lefort V. Anisimova M. Hordijk W. Gascuel O. (2010). New algorithms and methods to estimate maximum-likelihood phylogenies: assessing the performance of PhyML 3.0. *Syst. Biol.* 59 307–321. 10.1093/sysbio/syq010 20525638

[B11] Jaillon O. Aury J.-M. Noel B. Policriti A. Clepet C. Casagrande A. (2007). The grapevine genome sequence suggests ancestral hexaploidization in major angiosperm phyla. *Nature* 449 463–467. 10.1038/nature06148 17721507

[B12] Jiao Y. Wickett N. J. Ayyampalayam S. Chanderbali A. S. Landherr L. Ralph P. E. (2011). Ancestral polyploidy in seed plants and angiosperms. *Nature* 473 97–100. 10.1038/nature09916 21478875

[B13] Jombart T. Kendall M. Almagro-Garcia J. Colijn C. (2017). Treespace: Statistical exploration of landscapes of phylogenetic trees. *Mol. Ecol. Resour.* 17 1385–1392. 10.1111/1755-0998.12676 28374552 PMC5724650

[B14] Kim C. Wang X. Lee T.-H. Jakob K. Lee G.-J. Paterson A. H. (2014). Comparative analysis of *Miscanthus* and *Saccharum* reveals a shared whole-genome duplication but different evolutionary fates. *Plant Cell* 26 2420–2429. 10.1105/tpc.114.125583 24963058 PMC4114942

[B15] Kumar S. Stecher G. Li M. Knyaz C. Tamura K. (2018). MEGA X: Molecular evolutionary genetics analysis across computing platforms. *Mol. Biol. Evol.* 35 1547–1549. 10.1093/molbev/msy096 29722887 PMC5967553

[B16] Larkin M. A. Blackshields G. Brown N. P. Chenna R. McGettigan P. A. McWilliam H. (2007). Clustal W and Clustal X version 2.0. *Bioinformatics* 23 2947–2948. 10.1093/bioinformatics/btm404 17846036

[B17] Liu S. Liu Y. Yang X. Tong C. Edwards D. Parkin I. A. P. (2014). The *Brassica oleracea* genome reveals the asymmetrical evolution of polyploid genomes. *Nat. Commun.* 5:3930. 10.1038/ncomms4930 24852848 PMC4279128

[B18] Lynch M. Conery J. S. (2000). The evolutionary fate and consequences of duplicate genes. *Science* 290 1151–1155. 10.1126/science.290.5494.1151 11073452

[B19] Lynch M. Force A. (2000). The probability of duplicate gene preservation by subfunctionalization. *Genetics* 154 459–473. 10629003 10.1093/genetics/154.1.459 PMC1460895

[B20] Ma S. Downard K. M. Wong J. W. H. (2017). Phylogenetic analysis using protein mass spectrometry. *Methods Mol. Biol.* 1549 135–146. 10.1007/978-1-4939-6740-7_11 27975289

[B21] Mondragon-Palomino M. Gaut B. S. (2005). Gene conversion and the evolution of three leucine-rich repeat gene families in *Arabidopsis thaliana*. *Mol. Biol. Evol.* 22 2444–2456. 10.1093/molbev/msi241 16120808

[B22] Mondragon-Palomino M. Meyers B. C. Michelmore R. W. Gaut B. S. (2002). Patterns of positive selection in the complete NBS-LRR gene family of *Arabidopsis thaliana*. *Genome Res.* 12 1305–1315. 10.1101/gr.159402 12213767 PMC186657

[B23] Nei M. Gojobori T. (1986). Simple methods for estimating the numbers of synonymous and nonsynonymous nucleotide substitutions. *Mol. Biol. Evol.* 3 418–426. 10.1093/oxfordjournals.molbev.a040410 3444411

[B24] Paterson A. H. Wendel J. F. Gundlach H. Guo H. Jenkins J. Jin D. (2012). Repeated polyploidization of *Gossypium* genomes and the evolution of spinnable cotton fibres. *Nature* 492 423–427. 10.1038/nature11798 23257886

[B25] Saitou N. Nei M. (1987). The neighbor-joining method: a new method for reconstructing phylogenetic trees. *Mol. Biol. Evol.* 4 406–425. 10.1093/oxfordjournals.molbev.a040454 3447015

[B26] Salman-Minkov A. Sabath N. Mayrose I. (2016). Whole-genome duplication as a key factor in crop domestication. *Nat. Plants* 2:16115. 10.1038/nplants.2016.115 27479829

[B27] The Tomato Genome Consortium. (2012). The tomato genome sequence provides insights into fleshy fruit evolution. *Nature* 485 635–641. 10.1038/nature11119 22660326 PMC3378239

[B28] Thompson J. D. Higgins D. G. Gibson T. J. (1994). CLUSTAL W: improving the sensitivity of progressive multiple sequence alignment through sequence weighting, position-specific gap penalties and weight matrix choice. *Nucleic Acids Res.* 22 4673–4680. 10.1093/nar/22.22.4673 7984417 PMC308517

[B29] van de Peer Y. Maere S. Meyer A. (2009). The evolutionary significance of ancient genome duplications. *Nat. Rev. Genet.* 10 725–732. 10.1038/nrg2600 19652647

[B30] Wang J. Sun P. Li Y. Liu Y. Yu J. Ma X. (2017). Hierarchically aligning 10 legume genomes establishes a family-level genomics platform. *Plant Physiol.* 174 284–300. 10.1104/pp.16.01981 28325848 PMC5411148

[B31] Wang J. Yuan J. Yu J. Meng F. Sun P. Li Y. (2019). Recursive paleohexaploidization shaped the durian genome. *Plant Physiol.* 179 209–219. 10.1104/pp.18.00921 30385647 PMC6324235

[B32] Wang X. Guo H. Wang J. Lei T. Liu T. Wang Z. (2016). Comparative genomic de-convolution of the cotton genome revealed a decaploid ancestor and widespread chromosomal fractionation. *New Phytol.* 209 1252–1263. 10.1111/nph.13689 26756535

[B33] Wang X. Jin D. Wang Z. Guo H. Zhang L. Wang L. (2015). Telomere-centric genome repatterning determines recurring chromosome number reductions during the evolution of eukaryotes. *New Phytol.* 205 378–389. 10.1111/nph.12985 25138576

[B34] Wang X. Wang H. Wang J. Sun R. Wu J. Liu S. (2011). The genome of the mesopolyploid crop species *Brassica rapa*. *Nat. Genet.* 43 1035–1039. 10.1038/ng.919 21873998

[B35] Yang Z. (1994). Maximum likelihood phylogenetic estimation from DNA sequences with variable rates over sites: approximate methods. *J. Mol. Evol.* 39 306–314. 10.1007/bf00160154 7932792

[B36] Yang Z. (1997). PAML: a program package for phylogenetic analysis by maximum likelihood. *Comput. Appl. Biosci.* 13 555–556. 10.1093/bioinformatics/13.5.555 9367129

[B37] Yang Z. Nielsen R. Goldman N. Pedersen A. M. (2000). Codon-substitution models for heterogeneous selection pressure at amino acid sites. *Genetics* 155 431–449. 10790415 10.1093/genetics/155.1.431 PMC1461088

[B38] Yang Z. Rannala B. (2012). Molecular phylogenetics: principles and practice. *Nat. Rev. Genet.* 13 303–314. 10.1038/nrg3186 22456349

[B39] Yu J. Jung S. Cheng C.-H. Ficklin S. P. Lee T. Zheng P. (2014). CottonGen: a genomics, genetics and breeding database for cotton research. *Nucleic Acids Res.* 42 D1229–D1236. 10.1093/nar/gkt1064 24203703 PMC3964939

[B40] Zhang D. Kan X. Huss S. E. Jiang L. Chen L.-Q. Hu Y. (2018). Using phylogenetic analysis to investigate eukaryotic gene origin. *J. Vis. Exp.* 138:e56684. 10.3791/56684 30175990 PMC6126798

