# Supplementary material for: Cotton Duplicated Genes Produced by Polyploidy Show Significantly Elevated and Unbalanced Evolutionary Rates, Overwhelmingly Perturbing Gene Tree Topology
Source: Front Genet. 2020 Apr 23;11:239. doi: 10.3389/fgene.2020.00239 (PMC7190988; doi:10.3389/fgene.2020.00239)
Supplement: Supplementary file 1 [file Data_Sheet_1.PDF]

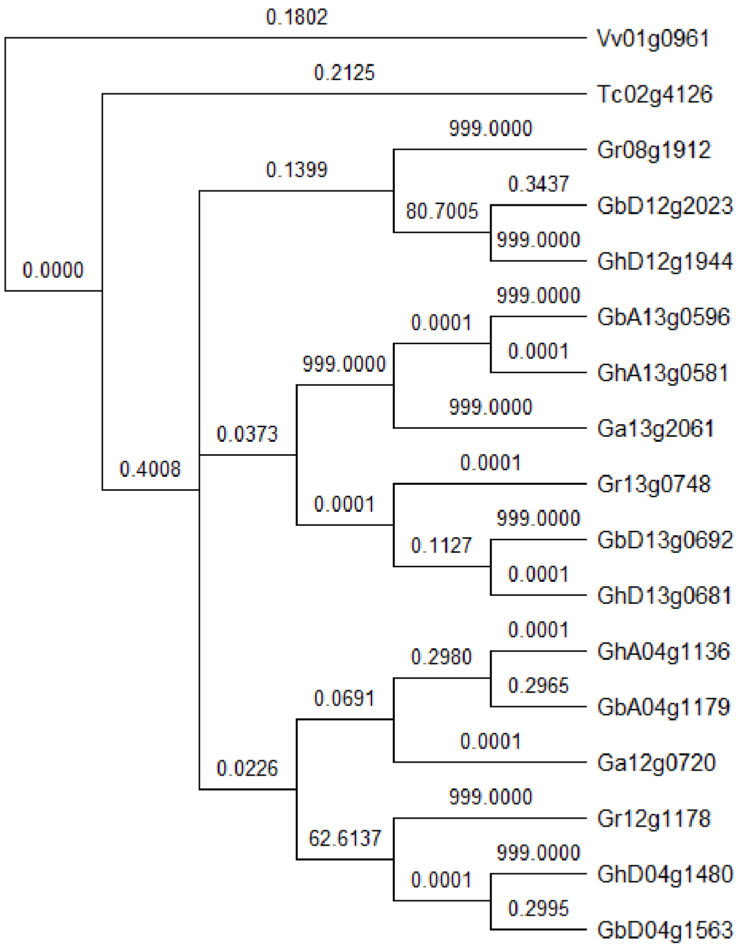
0.1802
Vv01g0961
0.2125
Tc02g4126
999.0000
Gr08g1912
0.1399
0.3437
80.7005
GbD12g2023
999.0000
0.0000
GhD12g1944
999.0000
0.0001
GbA13g0596
0.0001
999.0000
GhA13g0581
999.0000
Ga13g2061
0.0373
0.4008
0.0001
Gr13g0748
0.0001
999.0000
GbD13g0692
0.1127
0.0001
GhD13g0681
0.0001
GhA04g1136
0.2980
0.2965
0.0691
GbA04g1179
0.0001
Ga12g0720
0.0226
999.0000
Gr12g1178
62.6137
999.0000
GhD04g1480
0.0001
0.2995
GbD04g1563

E 1

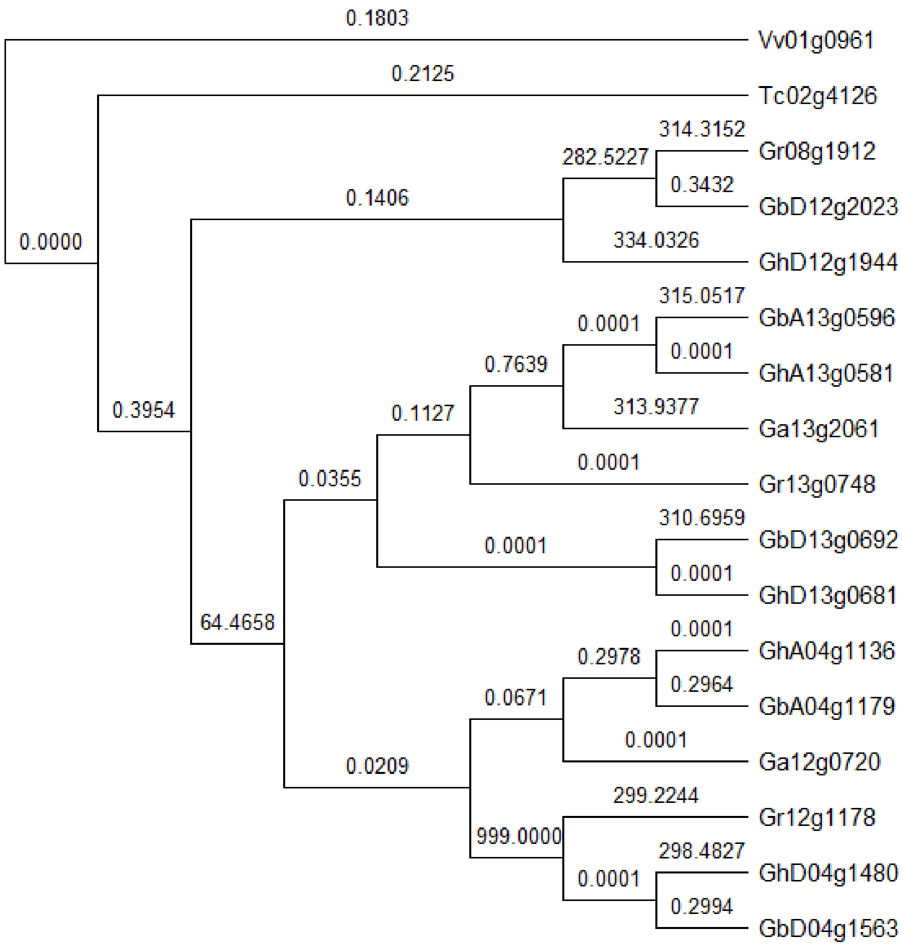
0.1803
Vv01g0961
0.2125
Tc02g4126
314.3152
282.5227
Gr08g1912
0.3432
0.1406
GbD12g2023
0.0000
334.0326
GhD12g1944
315.0517
0.0001
GbA13g0596
0.0001
0.7639
GhA13g0581
313.9377
0.3954
0.1127
Ga13g2061
0.0001
0.0355
Gr13g0748
310.6959
0.0001
GbD13g0692
0.0001
GhD13g0681
64.4658
0.0001
GhA04g1136
0.2978
0.2964
0.0671
GbA04g1179
0.0001
Ga12g0720
0.0209
299.2244
Gr12g1178
999.0000
298.4827
GhD04g1480
0.0001
0.2994
GbD04g1563

R 1

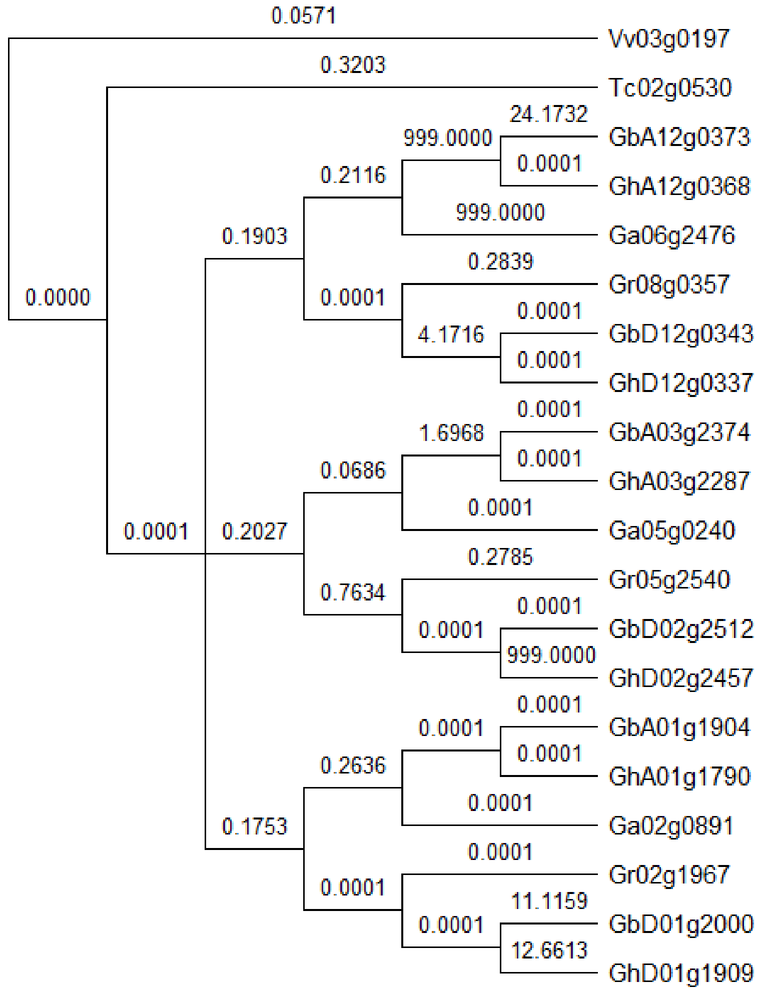
0.0571
Vv03g0197
0.3203
Tc02g0530
24.1732
999.0000
GbA12g0373
0.2116
0.0001
GhA12g0368
999.0000
0.1903
Ga06g2476
0.2839
0.0000
0.0001
Gr08g0357
0.0001
4.1716
GbD12g0343
0.0001
GhD12g0337
0.0001
1.6968
GbA03g2374
0.0686
0.0001
GhA03g2287
0.0001
0.0001
0.2027
Ga05g0240
0.2785
Gr05g2540
0.7634
0.0001
0.0001
GbD02g2512
999.0000
GhD02g2457
0.0001
0.0001
GbA01g1904
0.2636
0.0001
GhA01g1790
0.0001
0.1753
Ga02g0891
0.0001
Gr02g1967
0.0001
11.1159
0.0001
GbD01g2000
12.6613
GhD01g1909

E 2

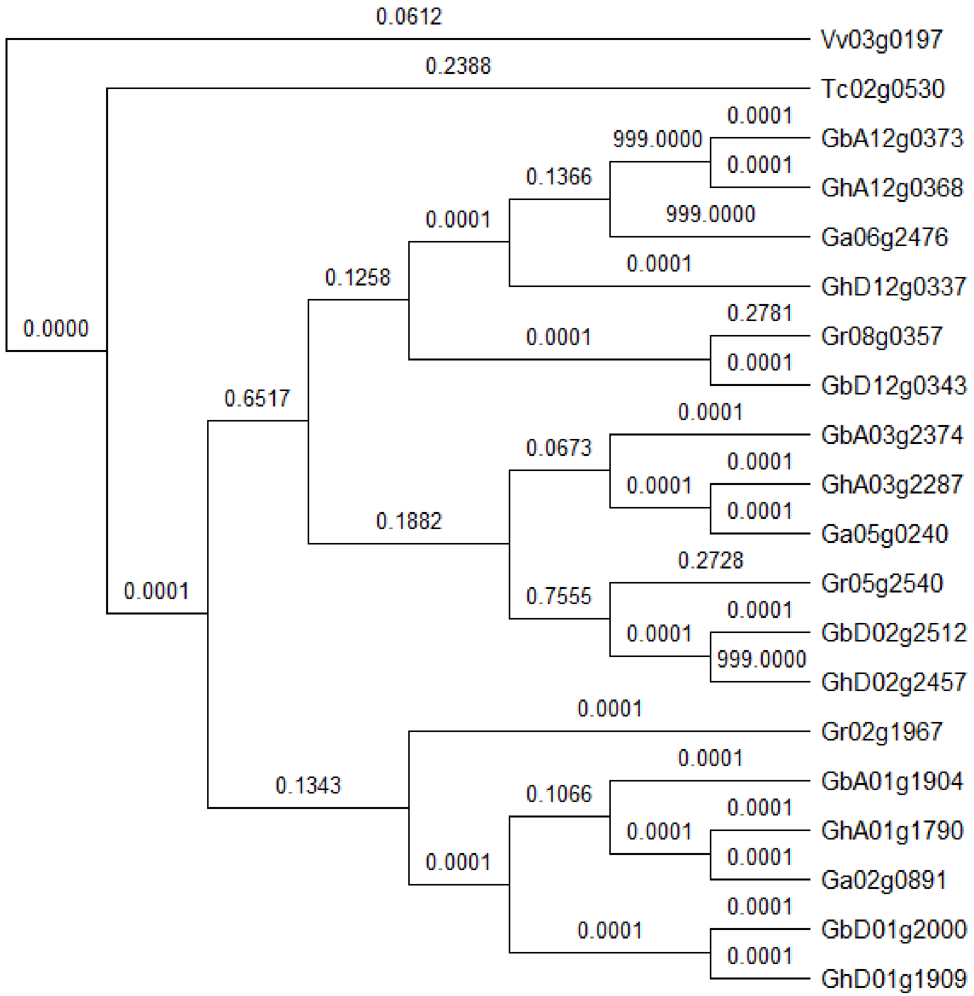
0.0612
Vv03g0197
0.2388
Tc02g0530
0.0001
999.0000
GbA12g0373
0.0001
0.1366
GhA12g0368
0.0001
999.0000
Ga06g2476
0.1258
0.0001
GhD12g0337
0.2781
0.0000
0.0001
Gr08g0357
0.0001
GbD12g0343
0.6517
0.0001
GbA03g2374
0.0673
0.0001
GhA03g2287
0.0001
0.0001
0.1882
Ga05g0240
0.2728
Gr05g2540
0.7555
0.0001
0.0001
0.0001
GbD02g2512
999.0000
GhD02g2457
0.0001
Gr02g1967
0.0001
GbA01g1904
0.1343
0.1066
0.0001
0.0001
GhA01g1790
0.0001
0.0001
Ga02g0891
0.0001
0.0001
GbD01g2000
0.0001
GhD01g1909

R 2

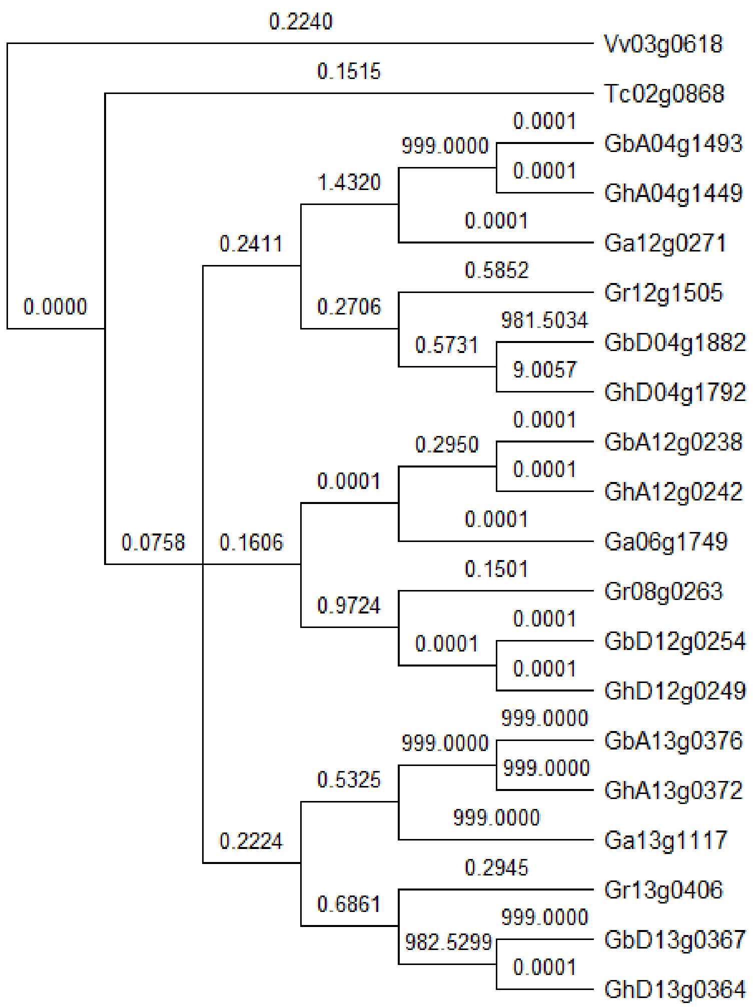

0.2240
Vv03g0618
0.1515
Tc02g0868
0.0001
999.0000
GbA04g1493
0.0001
GhA04g1449
1.4320
0.0001
Ga12g0271
0.2411
0.5852
Gr12g1505
0.0000
0.2706
981.5034
GbD04g1882
0.5731
9.0057
GhD04g1792
0.0001
0.2950
GbA12g0238
0.0001
GhA12g0242
0.0001
0.0001
Ga06g1749
0.0758
0.1606
0.1501
Gr08g0263
0.9724
0.0001
GbD12g0254
0.0001
0.0001
GhD12g0249
999.0000
999.0000
GbA13g0376
999.0000
GhA13g0372
0.5325
999.0000
Ga13g1117
0.2224
0.2945
Gr13g0406
0.6861
999.0000
982.5299
GbD13g0367
0.0001
GhD13g0364

E 3

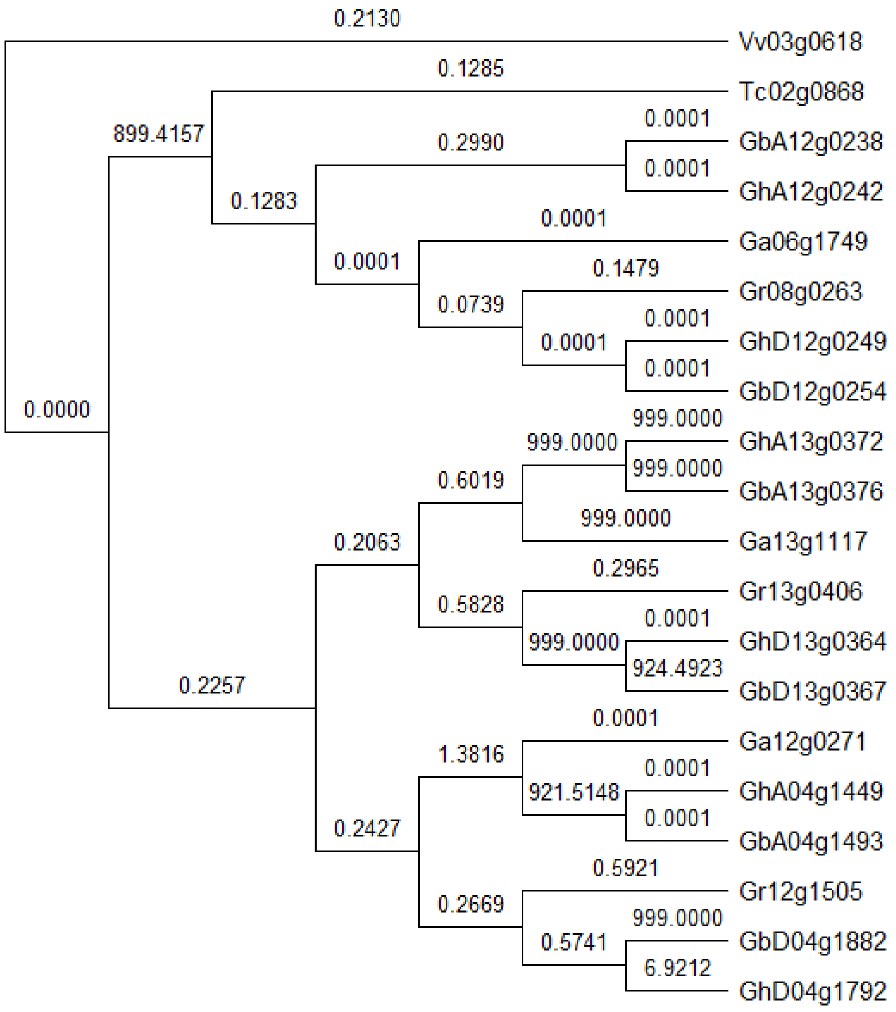

0.2130
Vv03g0618
0.1285
Tc02g0868
899.4157
0.2990
0.0001
GbA12g0238
0.0001
GhA12g0242
0.1283
0.0001
Ga06g1749
0.0001
0.1479
Gr08g0263
0.0739
0.0001
GhD12g0249
0.0001
0.0001
GbD12g0254
0.0000
999.0000
999.0000
GhA13g0372
999.0000
GbA13g0376
0.6019
999.0000
Ga13g1117
0.2063
0.2965
Gr13g0406
0.5828
0.0001
999.0000
GhD13g0364
924.4923
GbD13g0367
0.2257
0.0001
Ga12g0271
1.3816
0.0001
921.5148
GhA04g1449
0.0001
GbA04g1493
0.2427
0.5921
Gr12g1505
0.2669
999.0000
0.5741
GbD04g1882
6.9212
GhD04g1792

R 3

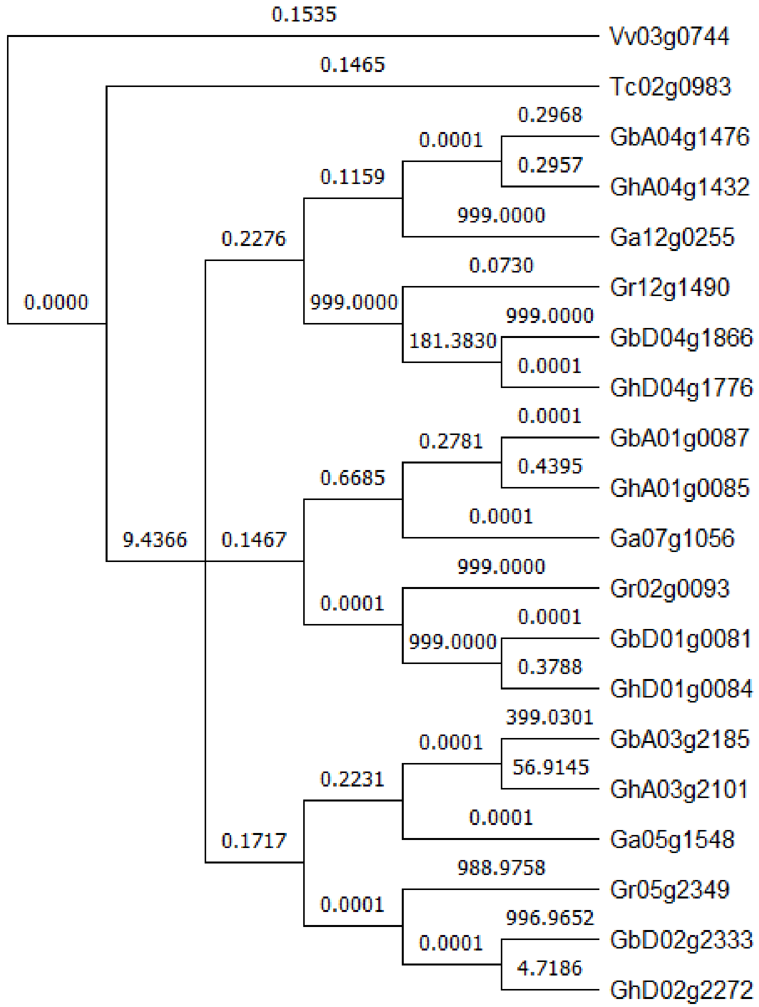
0.1535
Vv03g0744
0.1465
Tc02g0983
0.2968
GbA04g1476
0.0001
0.2957
GhA04g1432
0.1159
999.0000
Ga12g0255
0.2276
0.0730
Gr12g1490
0.0000
999.0000
999.0000
GbD04g1866
181.3830
0.0001
GhD04g1776
0.0001
GbA01g0087
0.2781
0.4395
GhA01g0085
0.6685
0.0001
Ga07g1056
9.4366
0.1467
999.0000
Gr02g0093
0.0001
0.0001
GbD01g0081
999.0000
0.3788
GhD01g0084
399.0301
GbA03g2185
0.0001
56.9145
GhA03g2101
0.2231
0.0001
Ga05g1548
0.1717
988.9758
Gr05g2349
0.0001
996.9652
GbD02g2333
0.0001
4.7186
GhD02g2272

E 4

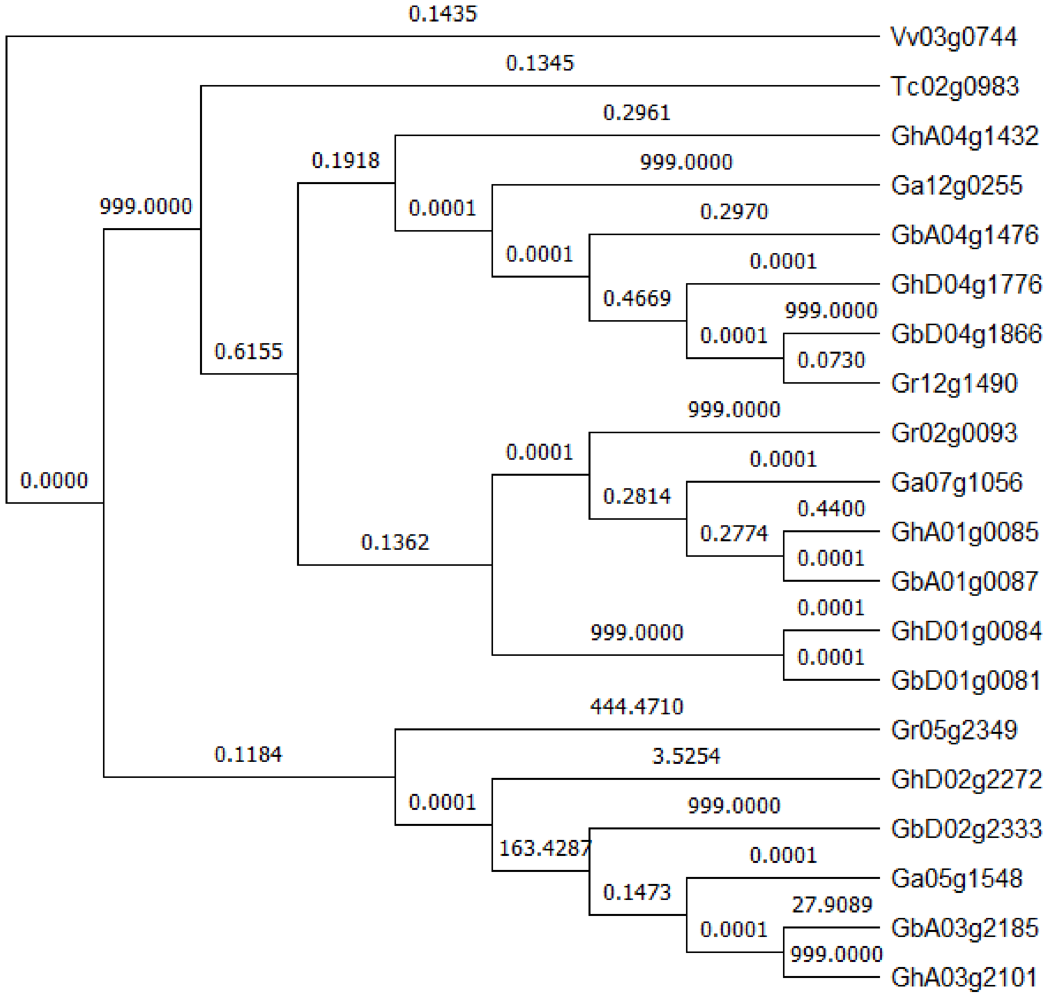
0.1435
Vv03g0744
0.1345
Tc02g0983
0.2961
GhA04g1432
0.1918
999.0000
Ga12g0255
999.0000
0.0001
0.2970
GbA04g1476
0.0001
0.0001
GhD04g1776
0.4669
999.0000
0.0001
GbD04g1866
0.6155
0.0730
Gr12g1490
999.0000
Gr02g0093
0.0001
0.0001
Ga07g1056
0.0000
0.2814
0.4400
0.2774
GhA01g0085
0.1362
0.0001
GbA01g0087
0.0001
999.0000
GhD01g0084
0.0001
GbD01g0081
444.4710
Gr05g2349
0.1184
3.5254
GhD02g2272
0.0001
999.0000
GbD02g2333
163.4287
0.0001
Ga05g1548
0.1473
27.9089
0.0001
GbA03g2185
999.0000
GhA03g2101

R 4

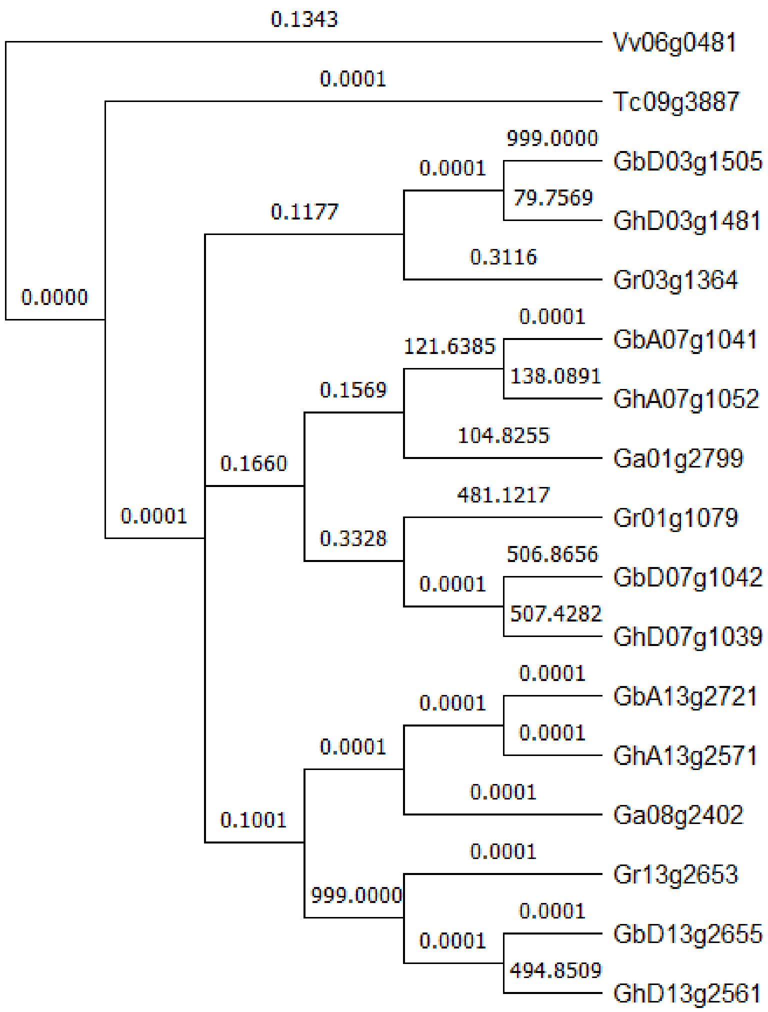
0.1343
Vv06g0481
0.0001
Tc09g3887
999.0000
0.0001
GbD03g1505
79.7569
GhD03g1481
0.1177
0.3116
Gr03g1364
0.0000
0.0001
GbA07g1041
121.6385
138.0891
GhA07g1052
0.1569
104.8255
Ga01g2799
0.1660
481.1217
Gr01g1079
0.0001
0.3328
506.8656
GbD07g1042
0.0001
507.4282
GhD07g1039
0.0001
GbA13g2721
0.0001
0.0001
GhA13g2571
0.0001
0.0001
Ga08g2402
0.1001
0.0001
Gr13g2653
999.0000
0.0001
GbD13g2655
0.0001
494.8509
GhD13g2561

E 5

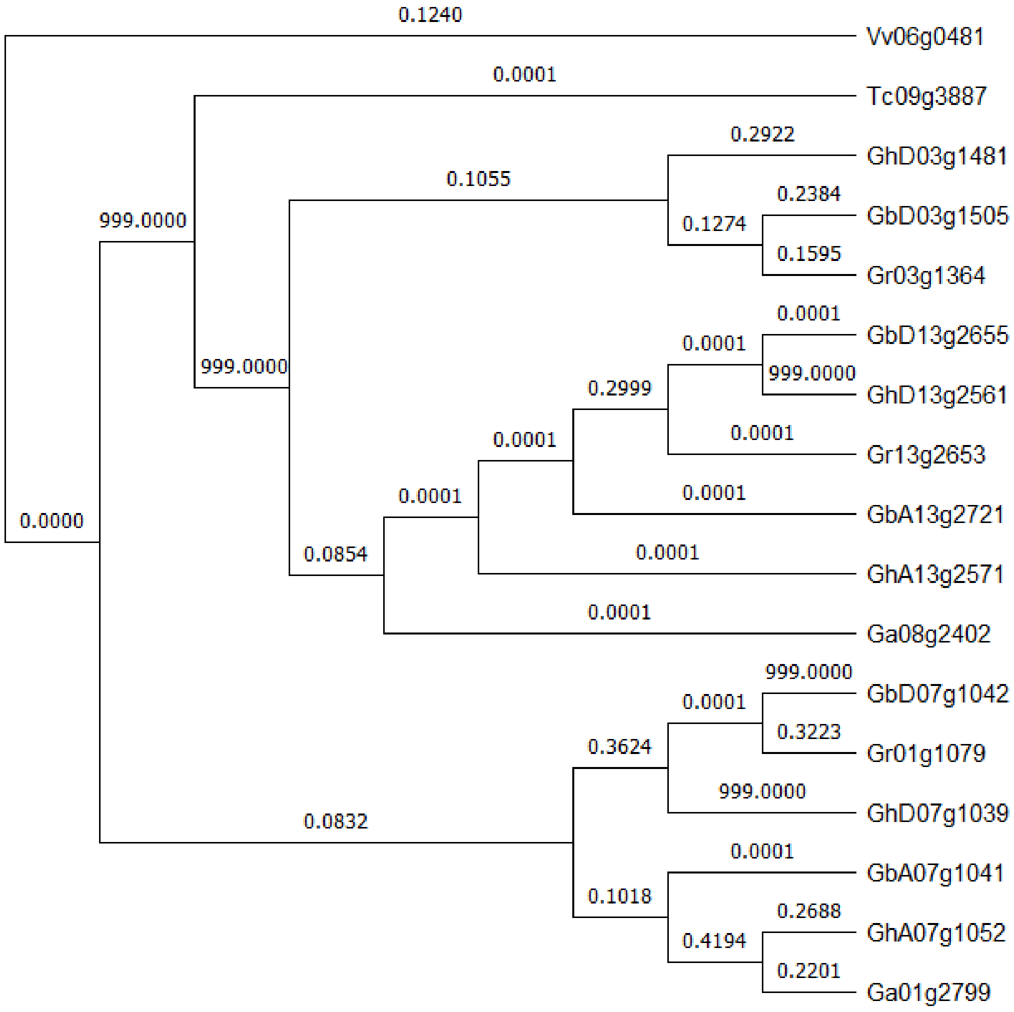
0.1240
Vv06g0481
0.0001
Tc09g3887
0.2922
GhD03g1481
0.1055
0.2384
GbD03g1505
999.0000
0.1274
0.1595
Gr03g1364
0.0001
GbD13g2655
0.0001
999.0000
999.0000
GhD13g2561
0.2999
0.0001
Gr13g2653
0.0001
0.0001
GbA13g2721
0.0000
0.0001
0.0854
0.0001
GhA13g2571
0.0001
Ga08g2402
999.0000
GbD07g1042
0.0001
0.3223
Gr01g1079
0.3624
999.0000
GhD07g1039
0.0832
0.0001
GbA07g1041
0.1018
0.2688
GhA07g1052
0.4194
0.2201
Ga01g2799

R 5

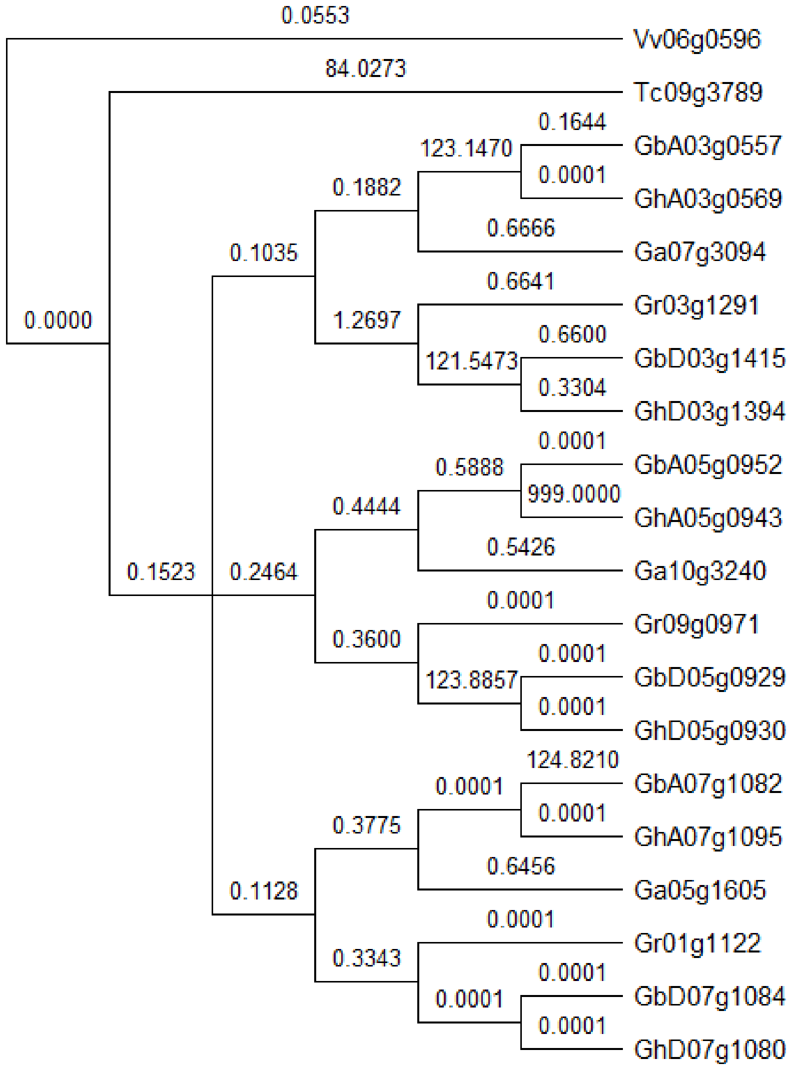
0.0553
Vv06g0596
84.0273
Tc09g3789
0.1644
123.1470
GbA03g0557
0.0001
0.1882
GhA03g0569
0.6666
0.1035
Ga07g3094
0.6641
0.0000
1.2697
Gr03g1291
0.6600
121.5473
GbD03g1415
0.3304
GhD03g1394
0.0001
0.5888
GbA05g0952
999.0000
0.4444
GhA05g0943
0.5426
0.1523
0.2464
Ga10g3240
0.0001
0.3600
Gr09g0971
0.0001
123.8857
GbD05g0929
0.0001
GhD05g0930
124.8210
0.0001
GbA07g1082
0.0001
0.3775
GhA07g1095
0.6456
0.1128
Ga05g1605
0.0001
0.3343
Gr01g1122
0.0001
0.0001
GbD07g1084
0.0001
GhD07g1080

E 6

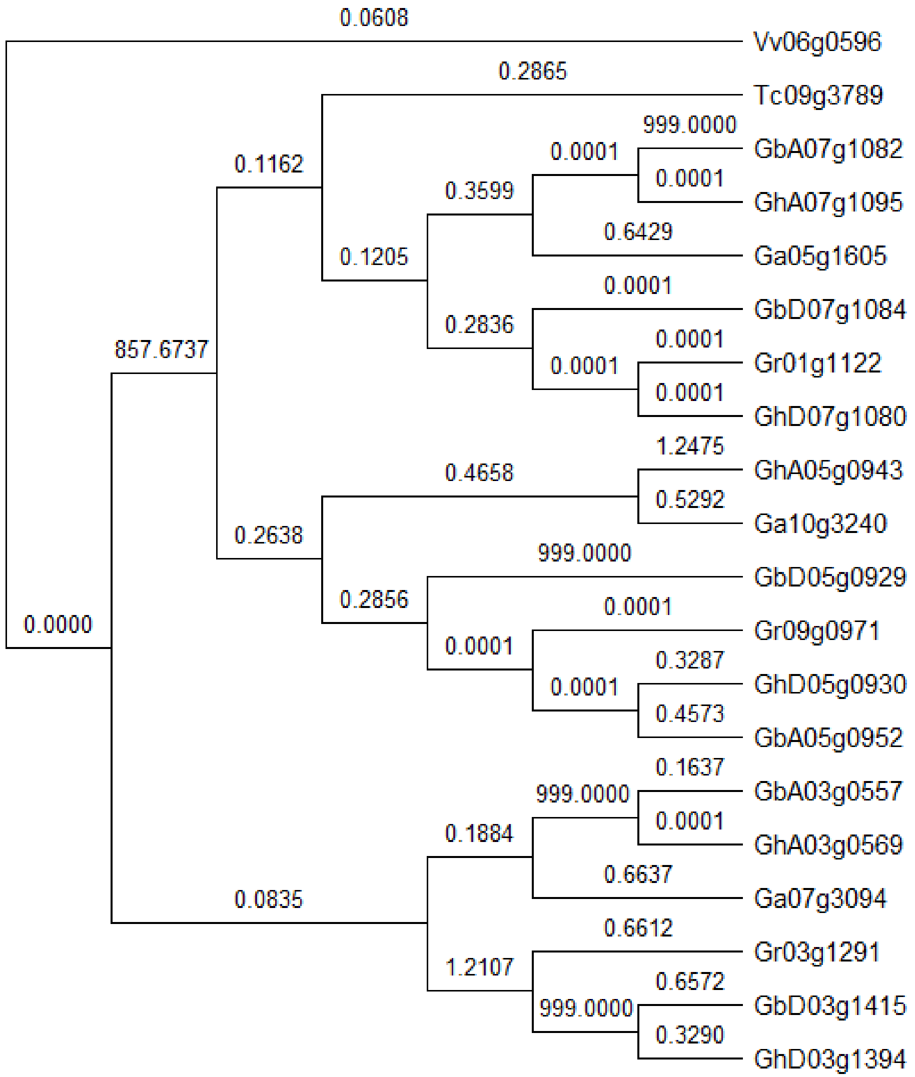
0.0608
Vv06g0596
0.2865
Tc09g3789
999.0000
0.0001
GbA07g1082
0.1162
0.0001
0.3599
GhA07g1095
0.6429
0.1205
Ga05g1605
0.0001
0.2836
GbD07g1084
0.0001
857.6737
0.0001
Gr01g1122
0.0001
GhD07g1080
1.2475
0.4658
GhA05g0943
0.5292
0.2638
Ga10g3240
999.0000
GbD05g0929
0.2856
0.0001
0.0000
0.0001
Gr09g0971
0.3287
0.0001
GhD05g0930
0.4573
GbA05g0952
0.1637
999.0000
GbA03g0557
0.0001
0.1884
GhA03g0569
0.0835
0.6637
Ga07g3094
0.6612
1.2107
Gr03g1291
0.6572
999.0000
GbD03g1415
0.3290
GhD03g1394

R 6

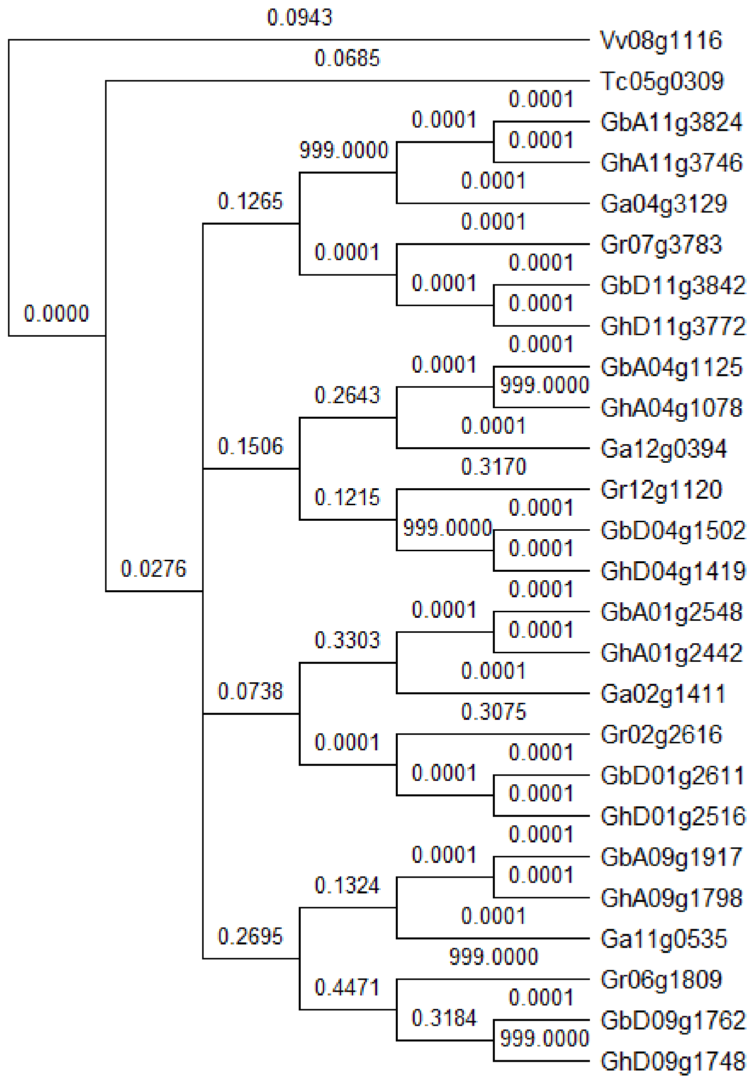

0.0943
Vv08g1116
0.0685
Tc05g0309
0.0001
0.0001
GbA11g3824
999.0000
0.0001
GhA11g3746
0.1265
0.0001
Ga04g3129
0.0001
Gr07g3783
0.0001
0.0001
GbD11g3842
0.0001
0.0001
GhD11g3772
0.0000
0.0001
0.0001
GbA04g1125
0.2643
999.0000
GhA04g1078
0.1506
0.0001
Ga12g0394
0.3170
Gr12g1120
0.1215
0.0001
999.0000
GbD04g1502
0.0001
GhD04g1419
0.0276
0.0001
0.0001
GbA01g2548
0.3303
0.0001
GhA01g2442
0.0738
0.0001
Ga02g1411
0.3075
Gr02g2616
0.0001
0.0001
0.0001
GbD01g2611
0.0001
GhD01g2516
0.0001
0.0001
GbA09g1917
0.1324
0.0001
GhA09g1798
0.2695
0.0001
Ga11g0535
999.0000
Gr06g1809
0.4471
0.0001
0.3184
GbD09g1762
999.0000
GhD09g1748

E 7

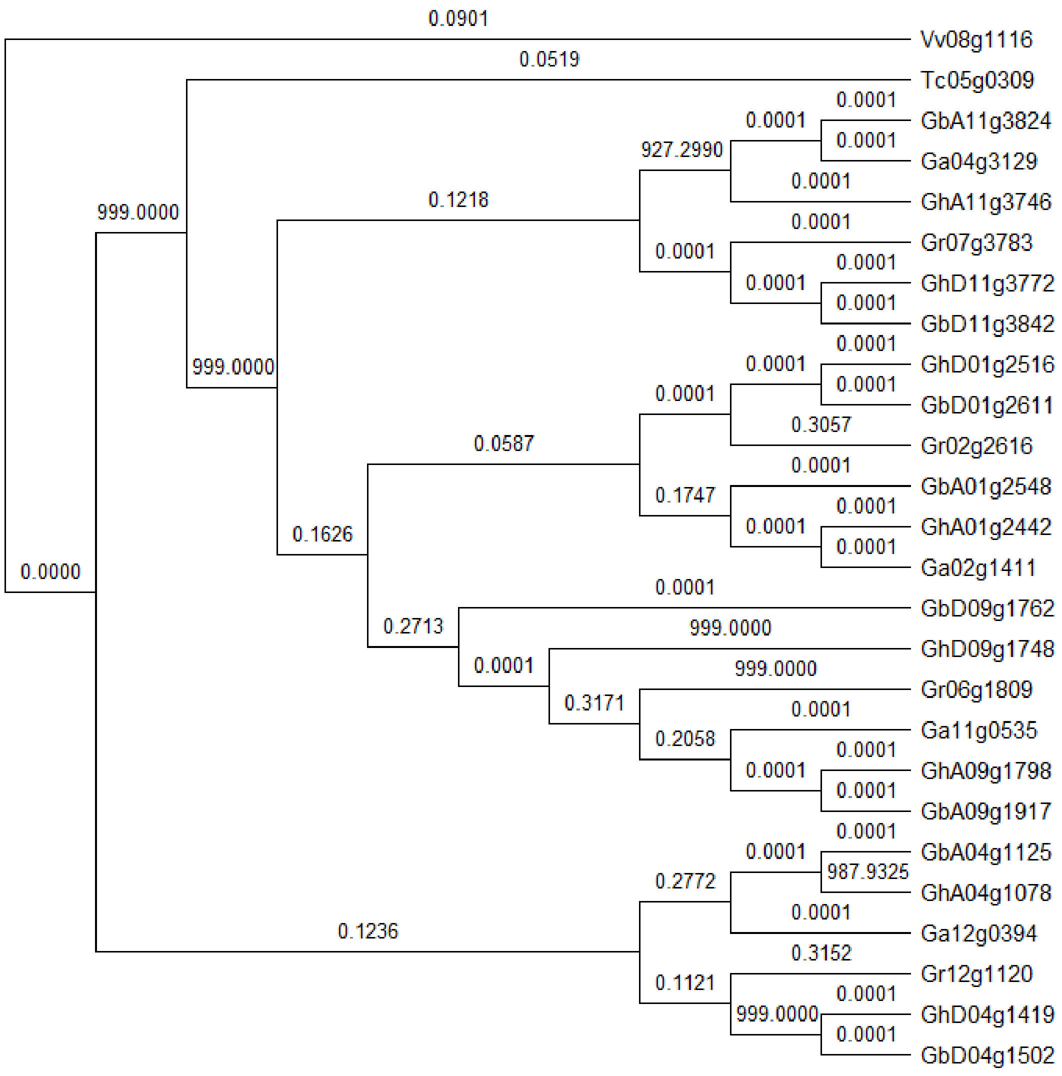

0.0901
Vv08g1116
0.0519
Tc05g0309
0.0001
0.0001
GbA11g3824
927.2990
0.0001
Ga04g3129
0.0001
GhA11g3746
0.1218
0.0001
Gr07g3783
999.0000
0.0001
0.0001
GhD11g3772
0.0001
0.0001
GbD11g3842
0.0001
GhD01g2516
0.0001
999.0000
0.0001
0.0001
GbD01g2611
0.3057
Gr02g2616
0.0587
0.0001
GbA01g2548
0.1747
0.0001
0.1626
0.0001
GhA01g2442
0.0001
Ga02g1411
0.0000
0.0001
GbD09g1762
0.2713
999.0000
GhD09g1748
0.0001
999.0000
Gr06g1809
0.3171
0.0001
Ga11g0535
0.2058
0.0001
0.0001
GhA09g1798
0.0001
GbA09g1917
0.0001
0.0001
GbA04g1125
0.2772
987.9325
GhA04g1078
0.0001
Ga12g0394
0.1236
0.3152
Gr12g1120
0.1121
0.0001
999.0000
GhD04g1419
0.0001
GbD04g1502

R 7

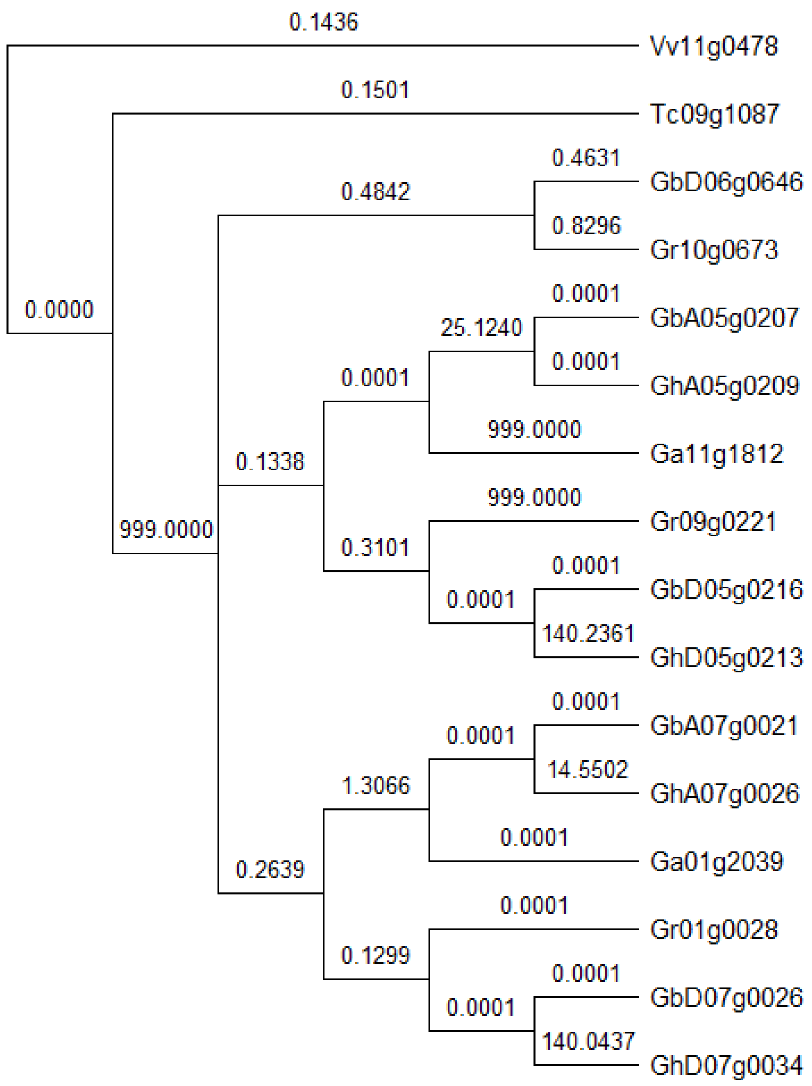

0.1436
Vv11g0478
0.1501
Tc09g1087
0.4631
GbD06g0646
0.4842
0.8296
Gr10g0673
0.0000
0.0001
GbA05g0207
25.1240
0.0001
GhA05g0209
0.0001
999.0000
Ga11g1812
0.1338
999.0000
Gr09g0221
999.0000
0.3101
0.0001
GbD05g0216
0.0001
140.2361
GhD05g0213
0.0001
GbA07g0021
0.0001
14.5502
GhA07g0026
1.3066
0.0001
Ga01g2039
0.2639
0.0001
Gr01g0028
0.1299
0.0001
GbD07g0026
0.0001
140.0437
GhD07g0034

E 8

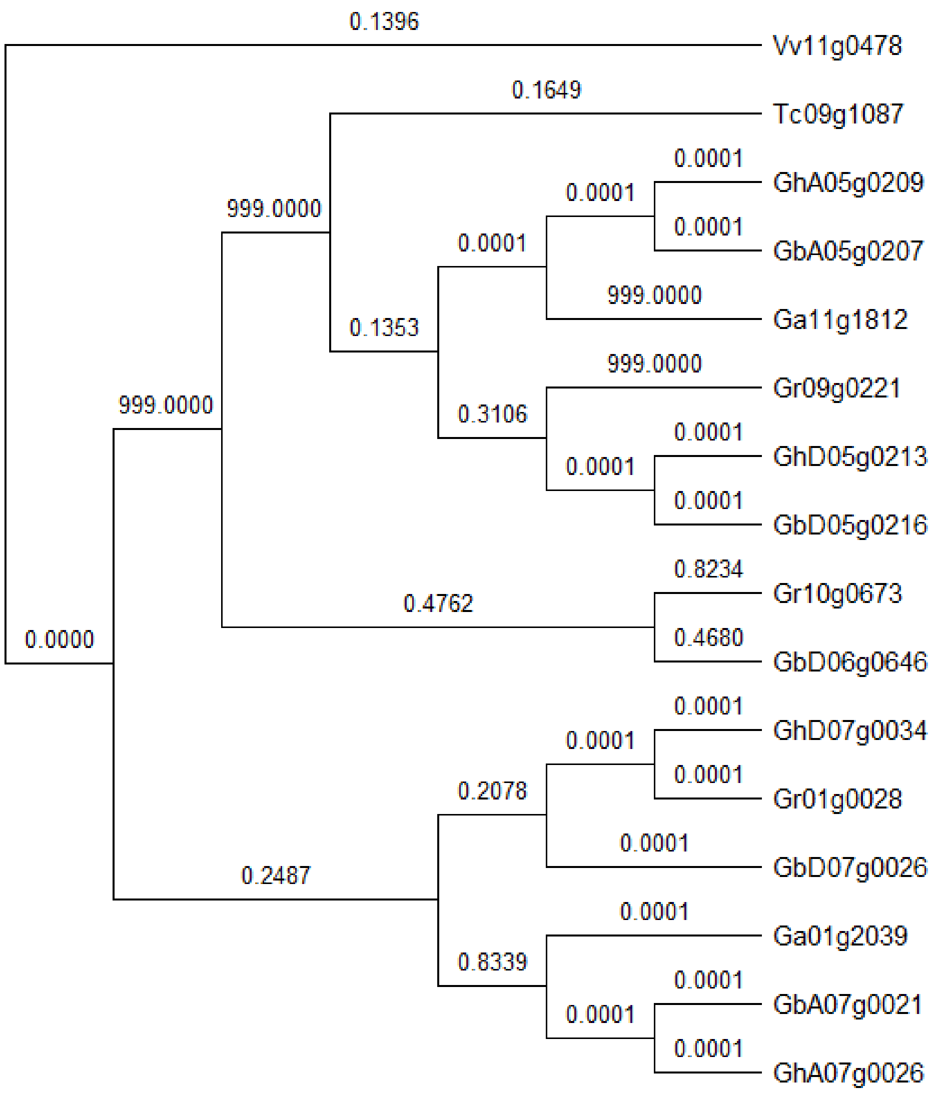

0.1396
Vv11g0478
0.1649
Tc09g1087
0.0001
GhA05g0209
0.0001
999.0000
0.0001
GbA05g0207
0.0001
999.0000
Ga11g1812
0.1353
999.0000
Gr09g0221
999.0000
0.3106
0.0001
GhD05g0213
0.0001
0.0001
GbD05g0216
0.8234
Gr10g0673
0.4762
0.0000
0.4680
GbD06g0646
0.0001
GhD07g0034
0.0001
0.0001
Gr01g0028
0.2078
0.0001
GbD07g0026
0.2487
0.0001
Ga01g2039
0.8339
0.0001
GbA07g0021
0.0001
0.0001
GhA07g0026

R 8

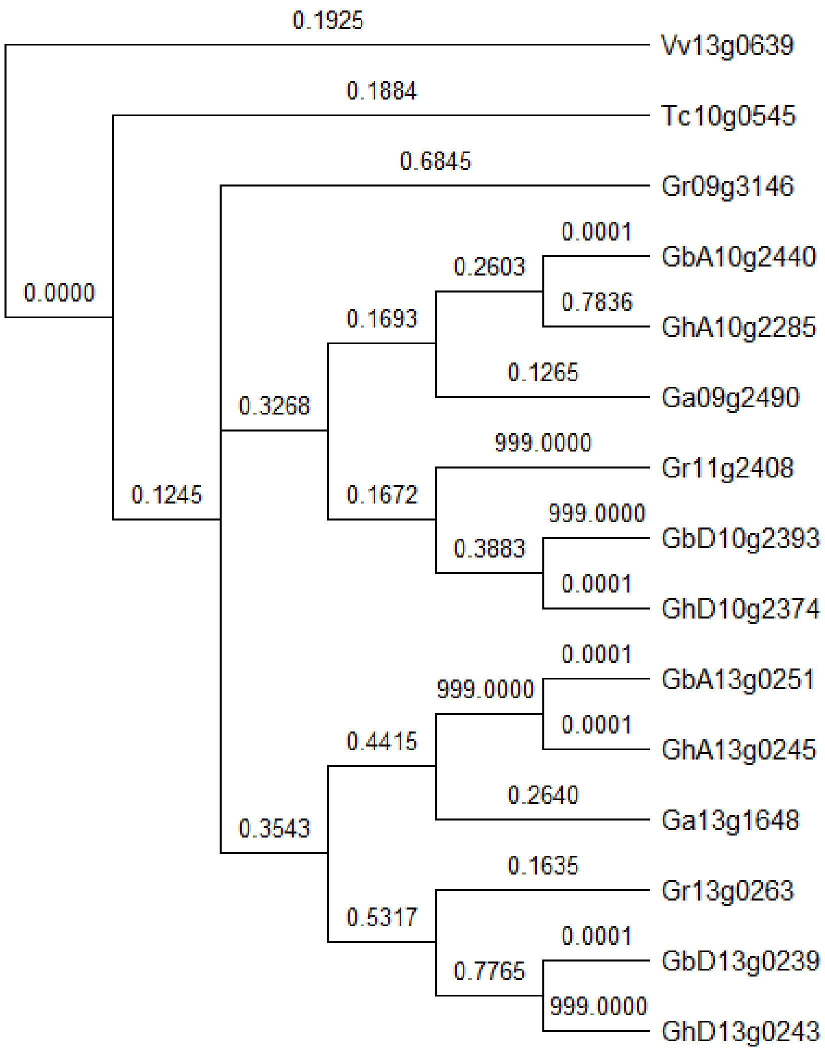
0.1925
Vv13g0639
0.1884
Tc10g0545
0.6845
Gr09g3146
0.0001
GbA10g2440
0.2603
0.7836
GhA10g2285
0.1693
0.1265
Ga09g2490
0.0000
0.3268
999.0000
Gr11g2408
0.1245
0.1672
999.0000
GbD10g2393
0.3883
0.0001
GhD10g2374
0.0001
GbA13g0251
999.0000
0.0001
GhA13g0245
0.4415
0.2640
Ga13g1648
0.3543
0.1635
Gr13g0263
0.5317
0.0001
GbD13g0239
0.7765
999.0000
GhD13g0243

E 9

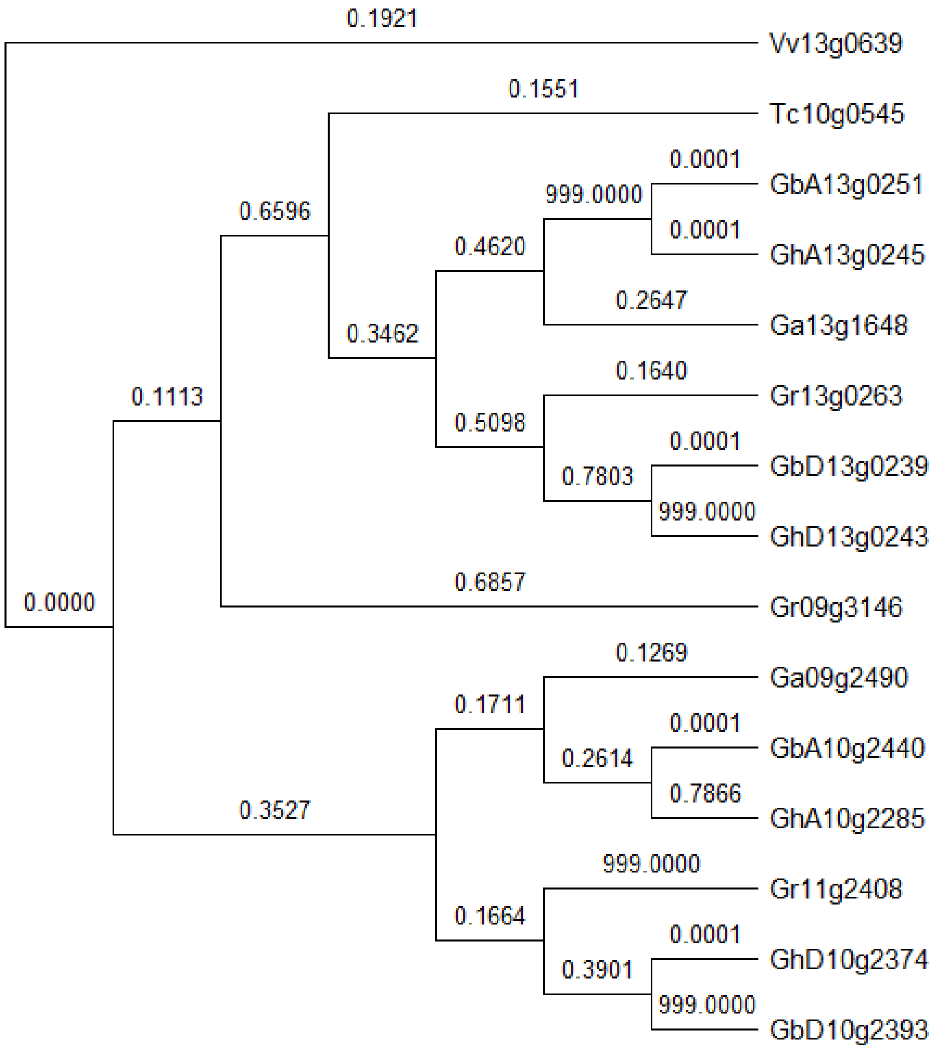
0.1921
Vv13g0639
0.1551
Tc10g0545
0.0001
GbA13g0251
999.0000
0.6596
0.0001
GhA13g0245
0.4620
0.2647
Ga13g1648
0.3462
0.1640
Gr13g0263
0.1113
0.5098
0.0001
GbD13g0239
0.7803
999.0000
GhD13g0243
0.0000
0.6857
Gr09g3146
0.1269
Ga09g2490
0.1711
0.0001
GbA10g2440
0.2614
0.7866
GhA10g2285
0.3527
999.0000
Gr11g2408
0.1664
0.0001
GhD10g2374
0.3901
999.0000
GbD10g2393

R 9

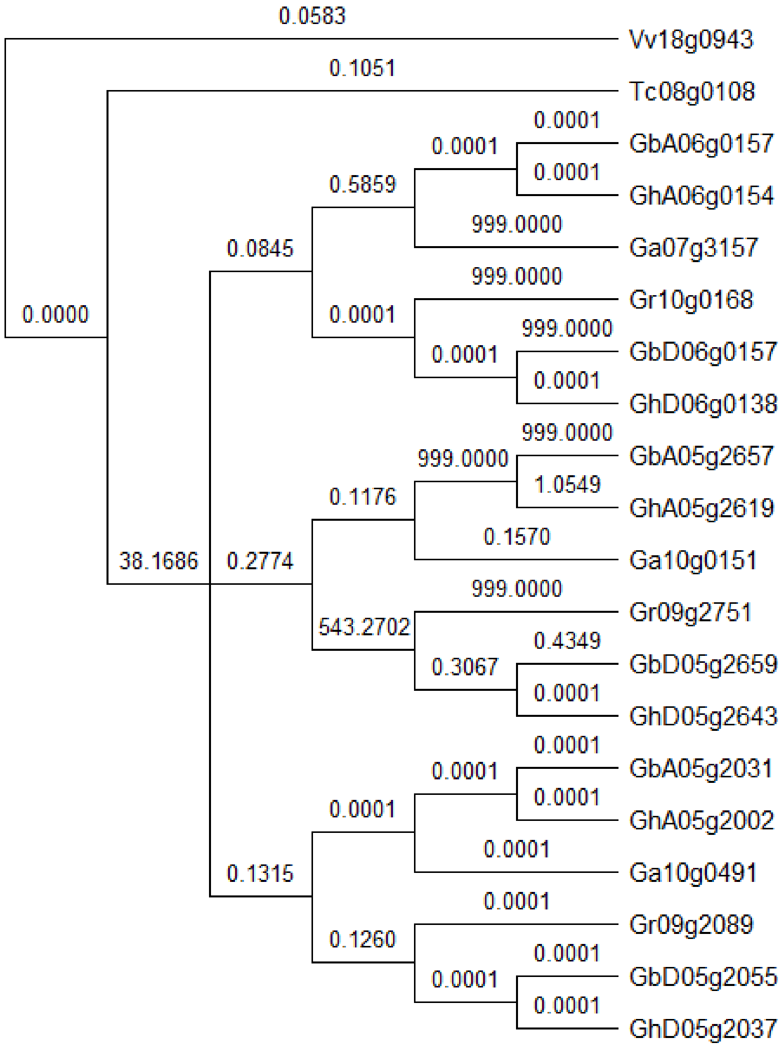
0.0583
Vv18g0943
0.1051
Tc08g0108
0.0001
GbA06g0157
0.0001
0.5859
0.0001
GhA06g0154
999.0000
Ga07g3157
0.0845
999.0000
Gr10g0168
0.0000
0.0001
999.0000
GbD06g0157
0.0001
0.0001
GhD06g0138
999.0000
GbA05g2657
999.0000
1.0549
GhA05g2619
0.1176
0.1570
Ga10g0151
38.1686
0.2774
999.0000
Gr09g2751
543.2702
0.4349
GbD05g2659
0.3067
0.0001
GhD05g2643
0.0001
GbA05g2031
0.0001
0.0001
0.0001
GhA05g2002
0.0001
Ga10g0491
0.1315
0.0001
Gr09g2089
0.1260
0.0001
GbD05g2055
0.0001
0.0001
GhD05g2037

E 10

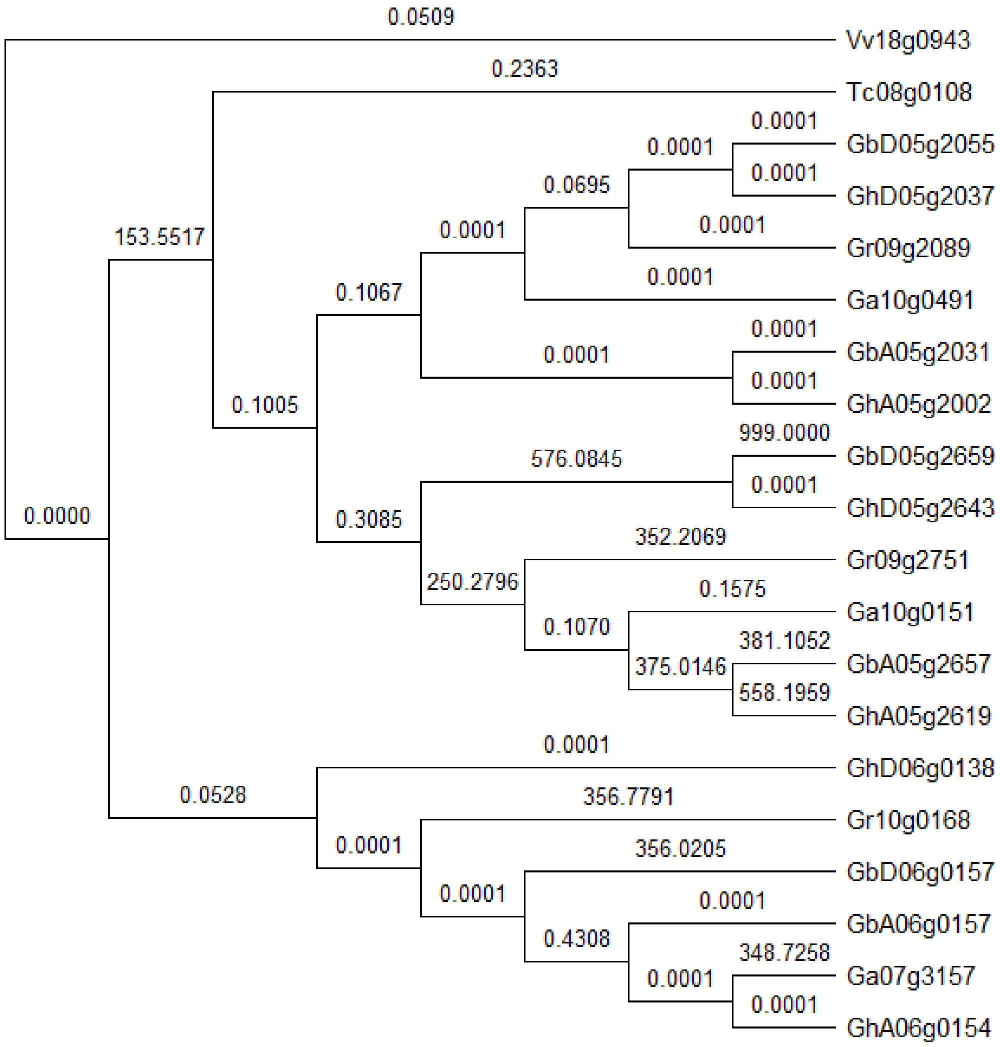
0.0509
Vv18g0943
0.2363
Tc08g0108
0.0001
GbD05g2055
0.0001
0.0001
GhD05g2037
0.0695
0.0001
Gr09g2089
153.5517
0.0001
0.0001
Ga10g0491
0.1067
0.0001
GbA05g2031
0.0001
0.0001
GhA05g2002
0.1005
999.0000
GbD05g2659
576.0845
0.0001
GhD05g2643
0.0000
0.3085
352.2069
Gr09g2751
250.2796
0.1575
Ga10g0151
0.1070
381.1052
GbA05g2657
375.0146
558.1959
GhA05g2619
0.0001
GhD06g0138
0.0528
356.7791
Gr10g0168
0.0001
356.0205
GbD06g0157
0.0001
0.0001
GbA06g0157
0.4308
348.7258
Ga07g3157
0.0001
0.0001
GhA06g0154

R 10

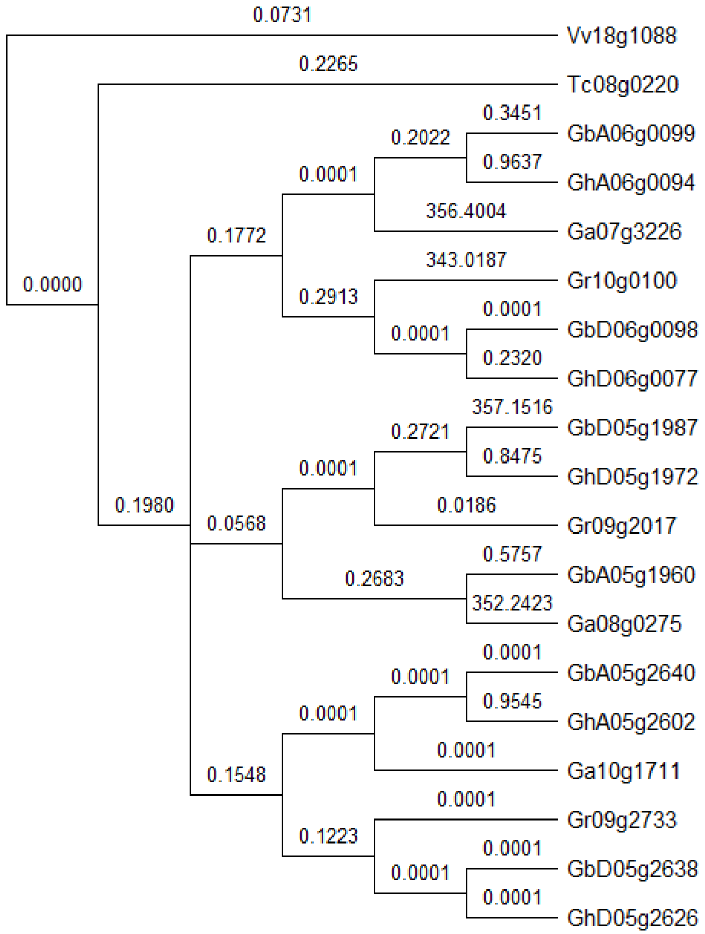
0.0731
Vv18g1088
0.2265
Tc08g0220
0.3451
GbA06g0099
0.2022
0.9637
GhA06g0094
0.0001
356.4004
Ga07g3226
0.1772
343.0187
Gr10g0100
0.0000
0.2913
0.0001
GbD06g0098
0.0001
0.2320
GhD06g0077
357.1516
GbD05g1987
0.2721
0.8475
GhD05g1972
0.0001
0.0186
Gr09g2017
0.1980
0.0568
0.5757
GbA05g1960
0.2683
352.2423
Ga08g0275
0.0001
GbA05g2640
0.0001
0.9545
GhA05g2602
0.0001
0.0001
Ga10g1711
0.1548
0.0001
Gr09g2733
0.1223
0.0001
GbD05g2638
0.0001
0.0001
GhD05g2626

E 11

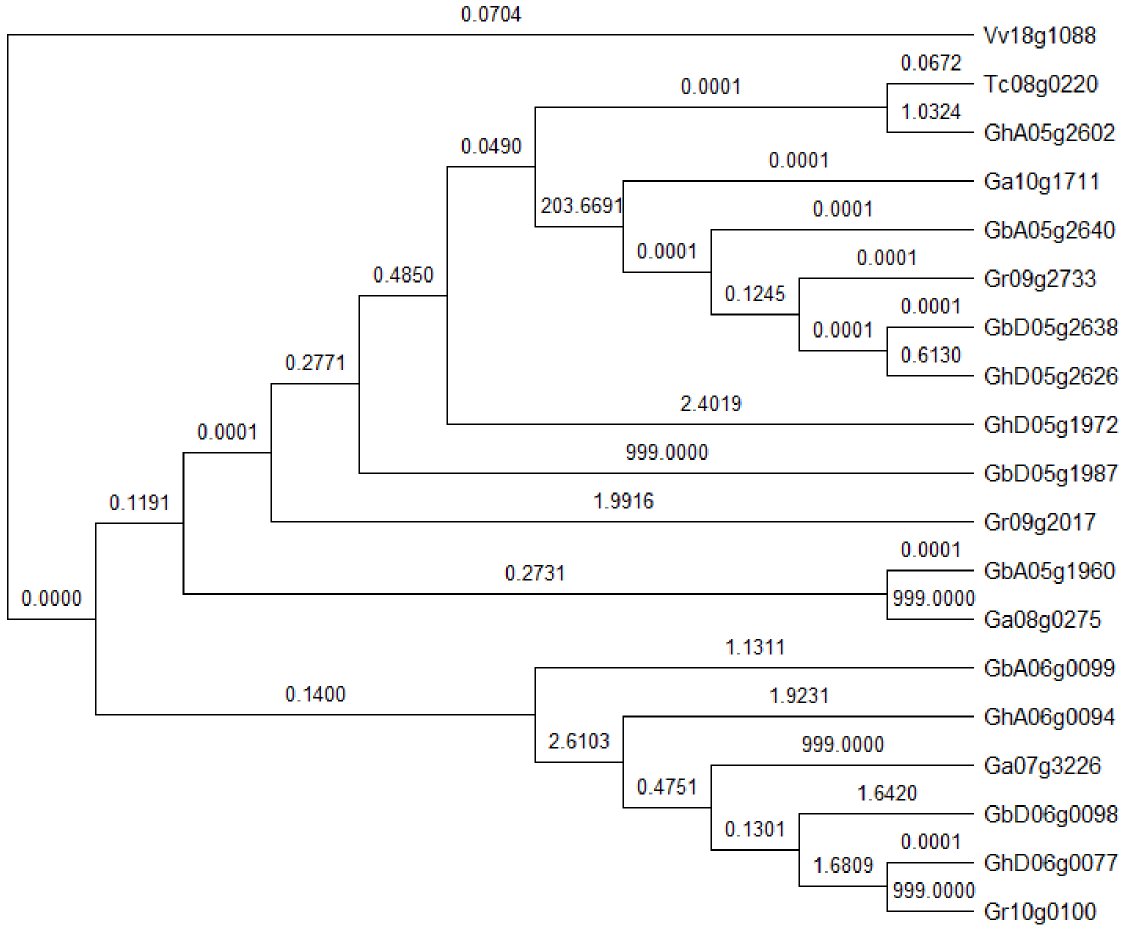
0.0704
Vv18g1088
0.0672
Tc08g0220
0.0001
1.0324
GhA05g2602
0.0490
0.0001
Ga10g1711
203.6691
0.0001
GbA05g2640
0.0001
0.0001
Gr09g2733
0.4850
0.1245
0.0001
GbD05g2638
0.0001
0.6130
GhD05g2626
0.2771
2.4019
GhD05g1972
0.0001
999.0000
GbD05g1987
0.1191
1.9916
Gr09g2017
0.0001
GbA05g1960
0.0000
0.2731
999.0000
Ga08g0275
1.1311
GbA06g0099
0.1400
1.9231
GhA06g0094
2.6103
999.0000
Ga07g3226
0.4751
1.6420
GbD06g0098
0.1301
0.0001
GhD06g0077
1.6809
999.0000
Gr10g0100

R 11

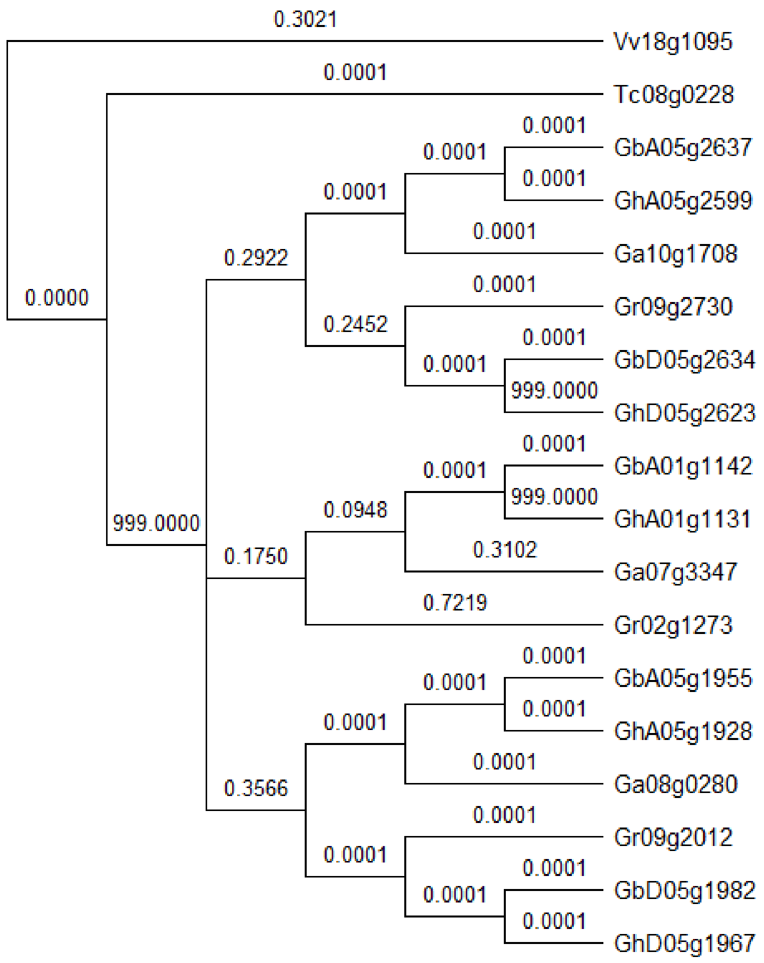
0.3021
Vv18g1095
0.0001
Tc08g0228
0.0001
0.0001
GbA05g2637
0.0001
GhA05g2599
0.0001
0.0001
Ga10g1708
0.2922
0.0000
0.0001
Gr09g2730
0.2452
0.0001
GbD05g2634
0.0001
999.0000
GhD05g2623
0.0001
0.0001
GbA01g1142
999.0000
GhA01g1131
0.0948
999.0000
0.3102
Ga07g3347
0.1750
0.7219
Gr02g1273
0.0001
0.0001
GbA05g1955
0.0001
GhA05g1928
0.0001
0.0001
Ga08g0280
0.3566
0.0001
Gr09g2012
0.0001
0.0001
GbD05g1982
0.0001
0.0001
GhD05g1967

E 12

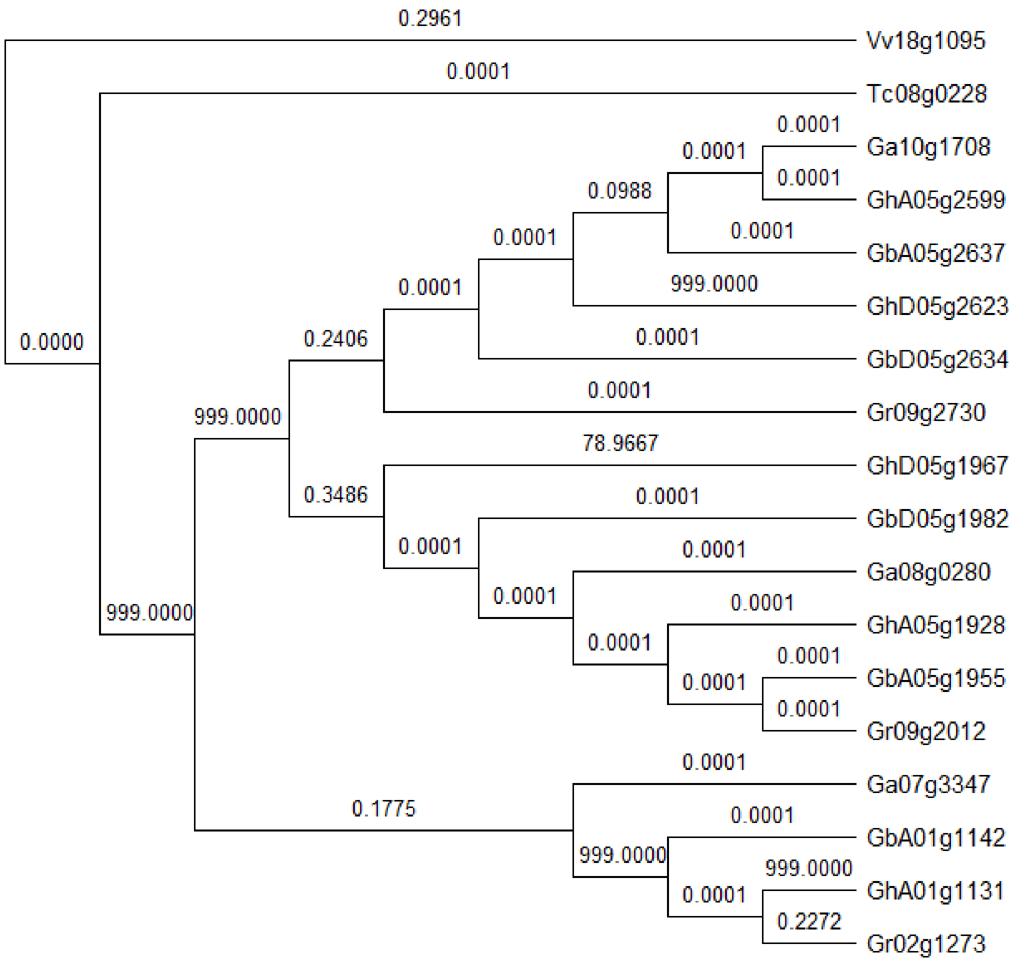
0.2961
Vv18g1095
0.0001
Tc08g0228
0.0001
0.0001
Ga10g1708
0.0988
0.0001
GhA05g2599
0.0001
0.0001
GbA05g2637
0.0001
999.0000
GhD05g2623
0.0001
GbD05g2634
0.2406
0.0000
0.0001
Gr09g2730
999.0000
78.9667
GhD05g1967
0.3486
0.0001
GbD05g1982
0.0001
0.0001
Ga08g0280
0.0001
0.0001
GhA05g1928
999.0000
0.0001
0.0001
GbA05g1955
0.0001
0.0001
Gr09g2012
0.0001
Ga07g3347
0.1775
0.0001
GbA01g1142
999.0000
999.0000
GhA01g1131
0.0001
0.2272
Gr02g1273

R 12

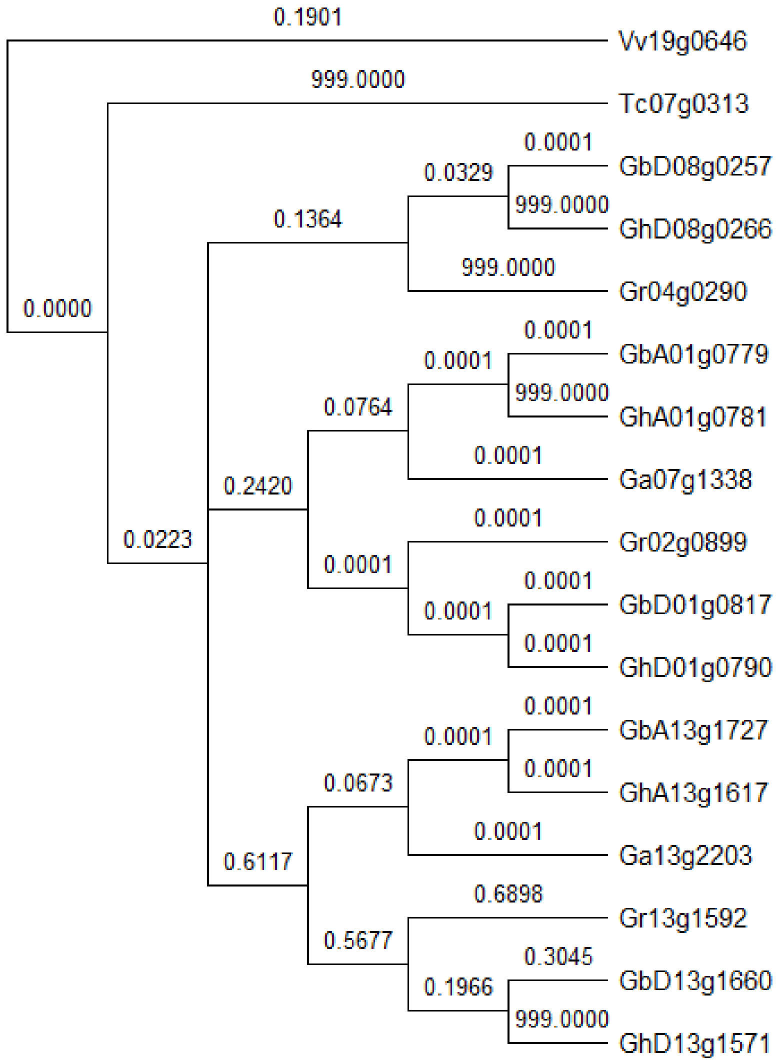

E 13

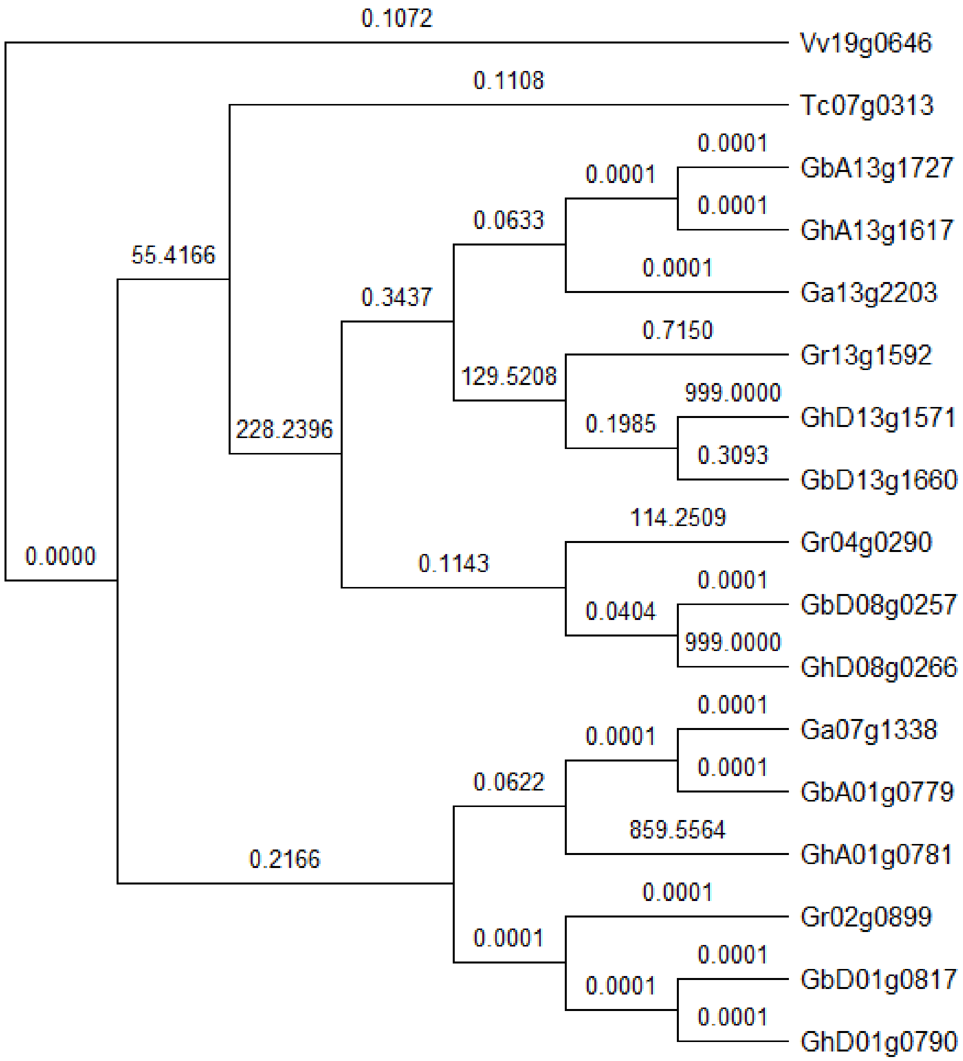

R 13

Figure S1. Natural selection of MYB genes in expected tree (E) and reconstructed tree (R) by branch model.
