# Supplementary material for: Cotton Duplicated Genes Produced by Polyploidy Show Significantly Elevated and Unbalanced Evolutionary Rates, Overwhelmingly Perturbing Gene Tree Topology
Source: Front Genet. 2020 Apr 23;11:239. doi: 10.3389/fgene.2020.00239 (PMC7190988; doi:10.3389/fgene.2020.00239)
Supplement: Supplementary file 2 [file Data_Sheet_2.zip › Data Sheet 2.zip/Table S3 - S4.DOCX]

***Supplementary Material***

# Supplementary Data

Supplementary Material includes Supplementary Tables S3 and S4. The left Supporting Tables (S1, S2 and S5) showed in supplemental excel tables. Supplementary Figures S1 showed in another supplemental PDF file.

# Supplementary Tables

**Table S3. Number of gene trees conforming to the expected topology using different methods after aligned by MUSCLE**.

| Method | Number | Percentage |
| --- | --- | --- |
| Maximum Likelihood | 109 | 16.5% |
| Neighbor-Joining | 101 | 15.3% |
| Minimum-Evolution | 107 | 16.2% |
| UPGMA | 6 | 0.9% |
| Maximum Parsimony | 99 | 15.0% |

**Table S4. Proportion of gene trees with expected topologies over different copy number ranges (E value cutoff of 1e-5)**.

| method | copy number | | | | |
| --- | --- | --- | --- | --- | --- |
|  | [0, 5） | [5, 10） | [10, 20） | [20, 50） | [50, 3113） |
| Maximum Likelihood | 22.2% | 15.7% | 18.0% | 19.9% | 12.8% |
| Neighbor-Joining | 20.0% | 14.3% | 16.5% | 17.3% | 10.1% |
| Minimum-Evolution | 20.0% | 17.9% | 17.3% | 19.2% | 10.1% |
| UPGMA | 2.2% | 0.7% | 1.5% | 0.6% | 0.5% |
| Maximum Parsimony | 6.7% | 13.6% | 14.3% | 15.4% | 11.7% |
